# Cytochalasins are a fascinating class of natural compounds: advances in the chemistry, biological activities, mode of action, and structure-activity relationships of novel cytochalasins isolated in the last decade are described

**DOI:** 10.1007/s13659-026-00651-x

**Published:** 2026-10-13

**Authors:** Antonio Evidente

**Affiliations:** Institute of Biomolecular Chemistry, Italian National Research Council (CNR), Viale Campi Flegrei 34, 80078 Pozzuoli, Italy

**Keywords:** Cytochalaisins, Covering period 2014–2024, Source, Chemical and biological characterization, Sructure activity relationships

## Abstract

Cytochalasins are a very intriguing group of natural compounds. Since the first isolation of the well-known cytochalasins A and B in 1966, over 300 analogues have been described as metabolites, primarily produced by various fungal genera. Their bioactivities, targets, total syntheses, and biosyntheses had been also extensively investigated. After the review of Scherlach et al., 2010 several other cytochalasins, isolated from different fungal sources, their biological activities and potential applications, were extensively described. In particular, during the last decades (2014–2024), as deduced from SciFinder research 259 articles, excluding reviews, were published essentially on new cytochalasins from new fungal sources and their biological activity and their potential application were reported. The reviews published in the same time (2014–2024) treated essentially the biological activities, the mode of action, the biosynthesis of already known cytochalasins and frequently the description was also extended and compared to other classes of natural compounds. The aims of the present review is to cover this gap reporting about the structure and biological activities of new cytochalasins isolated in the last decade (2014–2024) by fungi, endophytic fungi, essentially cohabitants in plants, and marine organisms. Furthermore, a brief section describing the cytochalasins Produced by lichens is also included. For some cytochalasins, the results of structure–activity and on the mode of action studies and potential biotechnological application will be also described.

**Graphical Abstract:**

**Figure:**
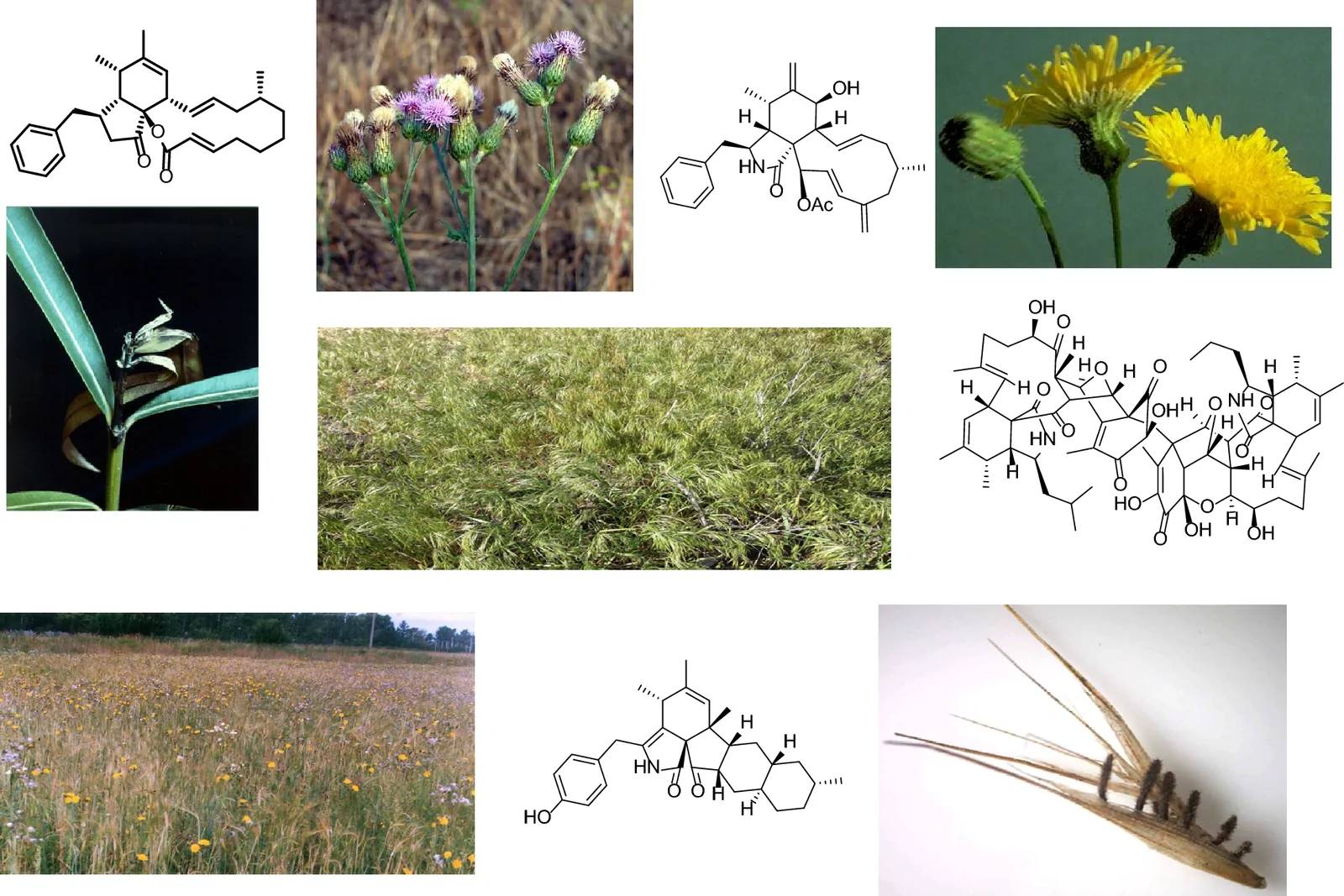

## Methods

### Search strategy

The search strategy focused on literature directly addressing the primary topic, while studies with less relevant content were excluded. Key electronic databases, search engines as Sci-Finder, Google, Google Scholar and Shi-Hub for articles meeting the inclusion standards. The search involved specific keyword combinations in titles and abstracts, using terms such as: (Cytochalasins, Fungi, Endophytes, Marine organisms, Likens and Biotechnological applications) This strategy yielded a broad selection of pertinent articles.

### Inclusion criteria

Publications from the search engines cited in Sect. 1.1 were selected based on their direct relevance to the research topic and their ability to provide accurate, complete and up-to-date data. The inclusion criteria referred to observational and experimental studies examining the origins of cytochalasins, their numerous biological activities, and structure–activity correlation studies, as well as potential practical applications, primarily in agriculture and medicine. Non-original publications (e.g., reviews, letters, or comments), redundant studies, and those lacking full-text availability or addressing unrelated conditions were excluded. Only studies published in English were considered, and copyright was respected through proper citation. This systematic and selective approach ensured that the analysis was grounded in credible scientific evidence and aligned with ethical research standards. The content of the article in each section and sub-section was chronologically reported.

## Introduction

Cytochalasins are a large group of natural metabolites, essentially produced by several fungal genera and some other organisms, and are well known for their cytotoxic activity on mammalian cells [1]. The name cytochalasins (cytos = cell, chalasis = relaxation) has been proposed as related at that time to their novel biological effects which were observed as above cited by Dr. Carter [1].

Cytochalasins are a group natural compounds which includes a diverse subgroup with a characteristic tricyclic core structure in which an 11 to 16-membered macrocycle ring, closed either a carbocycle, a lactone or a cyclic carbonate, is fused to a highly substituted perhydroisoindol-1-one [2]. Based on the l-amino acids incorporated into the hydrogenated isoindolone, the canonical cytochalasans are divided into different subgroups as cytochalasins (Phe), pyrichalasins (Tyr), aspochalasins (Leu), alachalasins (Ala) and chaetoglobosins (Trp) [3]. Usually, literature, including SciFinder source used to prepare this review, reported all together the compounds belonging to the first cited four groups as cytochalasins. Thus only chaetoglobusins are not treated in this review. The cytochalasins were also classified on the size of macrocyclic ring [3].

**Fig. 1:**
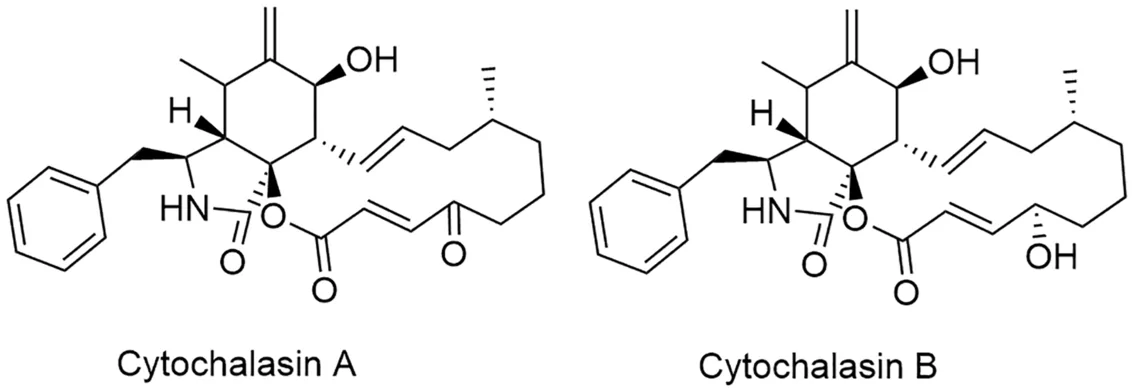
Structure of Cytochalasins A and B

The first two compounds belonging to this group and named cytochalasins A and B (Fig. 1), two 14-cytochalasans, were isolated at almost the same time by Rothweiler and Tam from *Phoma exigua* var. *exigua* [4, 5] and from Aldridge et al. [6], who purified the two cytochalasins from the culture filtrates of *Helminthosporium dematioideum* and *Metarrhizium anisopliae*. Cytochalasins C and D, two [11]-cytochalasans, were isolated from the same culture filtrates of the last two fungi [6].

Since that time several cytochalasins were essentially isolated by fungi and chemically and biological characterized. These cytochalasins, belonging to the different group of cytochalasans, were about 50 up to 1997 [7] and 300 more up to-day. Their bioactivities, targets, total syntheses, and biosyntheses had been also extensively investigated [3, 8–10]. After the review of Scherlach et al. [3], several other cytochalasins, which had been isolated from different fungal and other sources and their biological activities were extensively described. In particular, during the last decades (2014–2024), as deduced from SciFinder research, 259 articles, exluding reviews, were published. The other reviews, published in the same time (2014–2024), treated essentially the biological activities of already known cytochalasins, focusing on their therapeutic efficacy [11], their mode of action [12], their biosynthesis [10], and their use as chemotaxonomic markers [13]. Frequently, the description was also extended and compared to other classes of natural compounds. Just after this review was completed, Tammam et al. [14] published A review regarding cytochalasins over the period 2000–2023. Although this review cover ca. two decades. It is focused only fungal cytochalasins and in particular on their biosynthetic origins, pharmacokinetics and their promising therapeutic potential in drug discovery and development. In particular, their possible application in cardiovascular, oncology, cancer, inflammatory and autoimmune therapy was extensively treated. However, the approaches used by Tamman et al. is different from that used in the present one. They report in different sections an extensive decription on recent advances in biosynthesis, structural diversity and biological activities of cytochalasins derived from fungi. In particular, the main Sect. 3 is organized on the base of different class and subclass of fungal producer. The main deficiency of the review of Tamman et al. is that they decribed the chemical and biological characterization of on only ca. 10% of the new cytochalasins isolated in the last decade. Thus the aims of the present review is not only to cover this gap discussing the chemical and biological properties of all cytochalasins isolated from fungi (more than 300) but also those produced by marine organisms and other natural sources in the last decade (2014–2024). The very important role of cytochalasins to induced plants disease with economical severe consequence As well as their potential application as bioherbicides and in medicine towards different human diseases is also discussed. Furthermore, the results of several structure activity relationships studies (SAR) as well as some potential biotechnological applications of cytochalasins were also detailed described.

## Cytochalasins

### Cytochalasins from terrestrial fungi

The terrestrial fungi, including the plant pathogenic ones, are, among other microorganism, a very rich source of bioactive secondary metabolites belonging to several class of naturally occurring compounds and with a variety of potential application in different fields. Frequently, these kind of metabolites are also cytochalasins [15–19].

#### Cytochalasin from *Apergillus* spp.

Phytopathogenic fungi negatively affect plant growth and yield. Among them there are microrganisms belonging to the *Apergillus* genus and other genera which produce toxins that, in turn, accumulate in plants and lead to many problems, starting with the germination inhibition and disturbances in physiological processes [19] (Fig. 1).

##### Cytochalasins isolated from *Aspergillus* sp. PSU-RSPG185

**Fig. 2:**
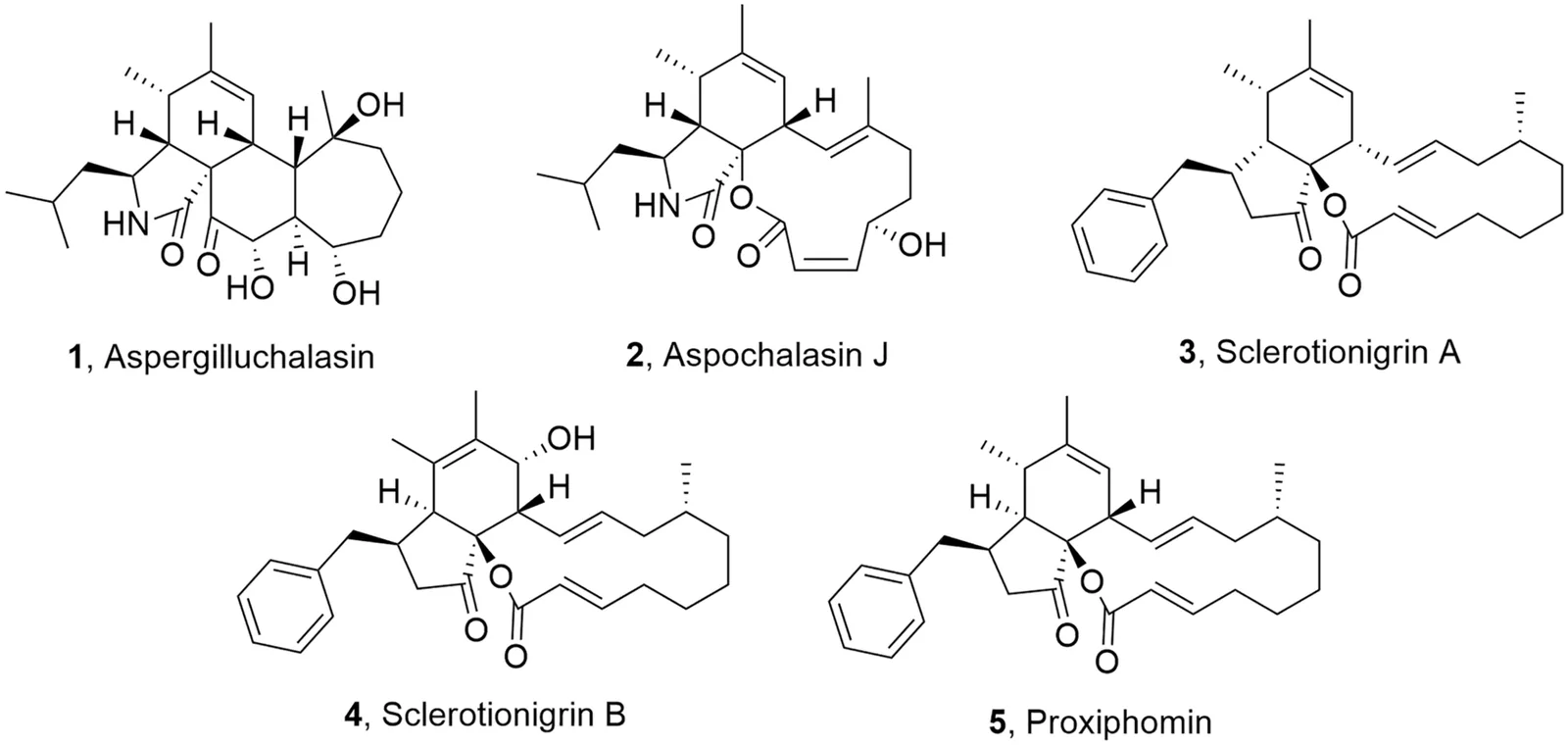
Cytochalasin isolated from *Aspergillus* sp. PSU-RSPG185 and *Aspergillus sclerotioniger *

The new aspergilluchalasin and aspochalasin J (**1** and **2**, Fig. 2) were isolated together with phenalenones, aspergillussanones A and B and aspergillulacton, two phenalenones and a γ-butyrolactone, respectively, from the mycelia and liquid culture of the soil fungus *Aspergillus* sp. PSU-RSPG185. From the same fungus were also isolated aspergillusols A and B, aspergillusic acid, two cyclic carbonate derivatives, a new eutypinic acid derivative, (+)-asperpentyn, 8,9-eutypinic acid, 4-hydroxy-3-prenylbenzoic acid, 2,2 dimethyl-2H-1-chromene-6-carboxylic acid and 2-(1′-methylethenyl)benzofuran-5-carboxylic. All the compounds were tested against KB and Vero cell lines and only aspergillussanones A showed weak activity with IC_50_ (Half Maximal Inhibitory Concentration**)** values of 48.4 and 34.2 μM, respectively. Furthermore, any metabolites showed antimalarial or antimycobacterial activity against *Plasmodium falciparum*, K1 strain or *Mycobacterium tuberculosis*, respectively [20].

##### Cytochalasins isolated from *Aspergillus sclerotioniger*

From another species of *Aspergillus*, *A. sclerotioniger,* sclerotionigrin A and B (**3** and **4**, Fig. 2) were isolated together with the known proxiphomin (**5**, Fig. 2). The cytotoxicity of compounds **3**–**5** against CLL (Chronic Lymphocytic Leukemia) cells was tested in vitro using a CellTiter-Glo^®^ assay. Compound **5** showed the strongest toxicity with LC_50_ (Lethal Concentration, 50%) values of ca. 48 μM whereas no effect was found towards healthy B-cells in concentrations < 100 μM. Compounds **3** exhibited minor activity at a concentration of 72 μM, while compound **4** was no toxic. This resulted indicated that the novel stereochemistry in the central ring system of compounds **3** and **4** negatively affected on target interactions [21].

##### Cytochalasin isolated from *Aspergillus flavipes*

**Fig. 3:**
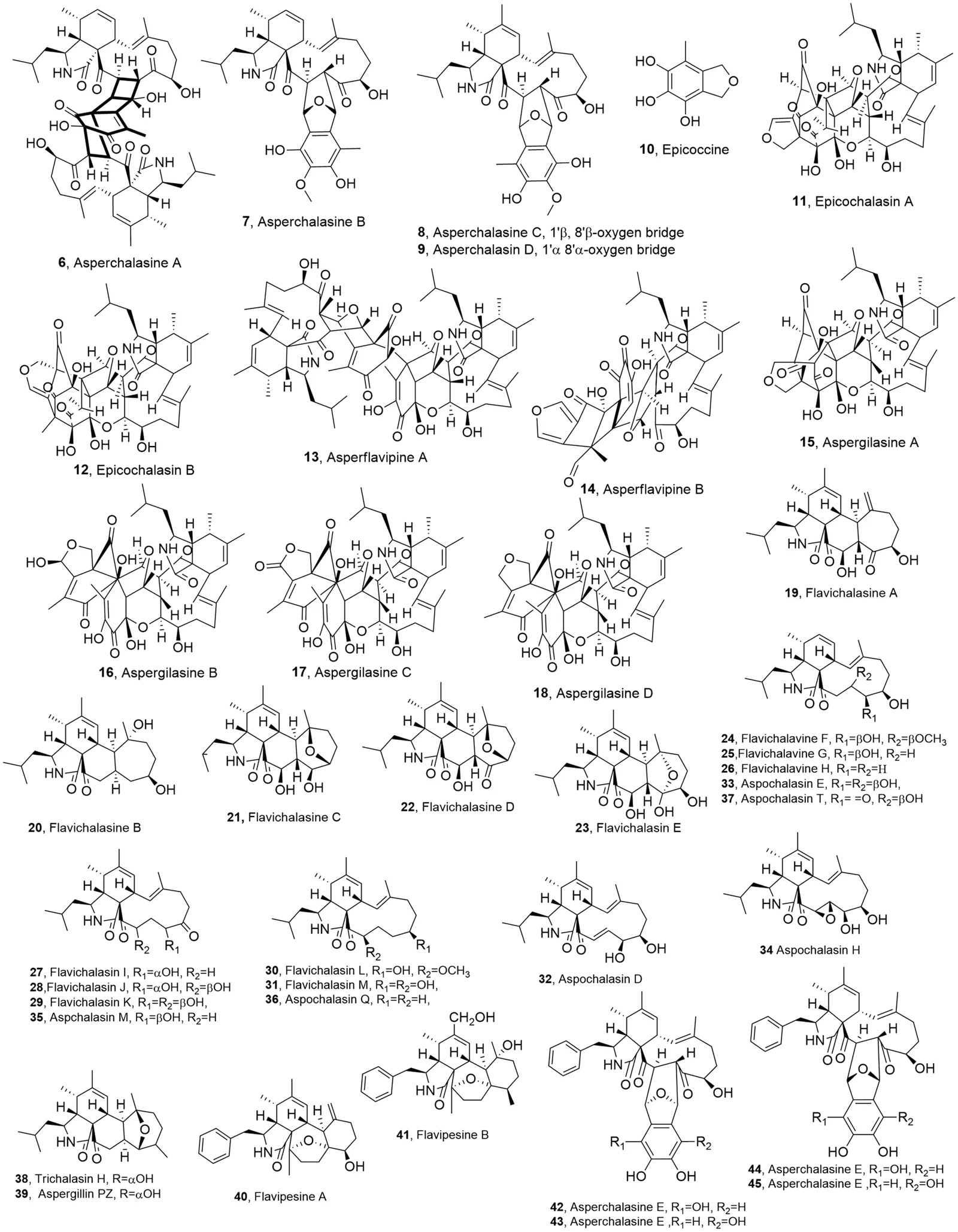
Cytochalasins isolated from *Aspergyllus flavipes *

From *Aspergillus flavipes* were isolated several new cytochalasins as asperchalasines A-D, (**6**–**9**, Fig. 3). Asperchalasine A (**6**) was the first example of a cytochalasan dimer generated by the fusion of two cytochalasan moieties to epicoccine (**10**, Fig. 3), resulting an unprecedented reported 13-oxatetracyclo [7.2.1.12, 5.01,6] tridec-8,12-dione core containing 20 chiral centers. Asperchalasines A-D (**7**–**9**) were assayed for their cytotoxic activity, and cytochalasin **9** was further studied for its cell cycle regulatory activity. In fact, it caused significant G1-phase cell cycle arrest in four cancer cell lines while have not effect on two normal cell lines [22]. Successively, epicochalasines A and B (**11** and **12**, Fig. 3), were isolated from the same fungus. These two cytochalasins were the first merocytochalasans bearing caged epicoccine (**10**, Fig. 3) dimer unit coupled with aspochalasin and this group was named epicochalasine. Compounds **11** and **12** were studied for their effect on the inhibition of proliferation of HL6 and NB4 leukemia cells and both epocochalasins showed significant G2/M phase cell arrest. Furthermore, cytochalasins **11** and **12** induced apoptosis in leukemia cells through the activation of caspase-3 and the degradation of PARP (Poly ADP-ribose polymerase) [23].

From the same strain QCS12 of *A. flavipes* collected in China asperflavipines A and B (**13** and **14**, Fig. 3), two new structurally complex merocytochalasans, were later isolated Compound **13**, which was the first dimeric cytochalasan, showed moderate cytotoxic towards seven cancer cell lines, with IC_50_ values ranging from 12.7 to 27.6 μM, while compound **14** was inactive up to 40 μM. Cytochalasin **13** induced apoptosis in these cells as was confirmed by successive flow cytometry analysis of cells treated. In addition, compound **13** altered the expression levels PARP and cleaved caspase-3. All these experiments were performed using Jurkat, NB4, and HL60 cells as models. Asperflavipines A (**13**) caused cell shrinking and severe blebbing of the plasma membrane suggesting that this cytochalasin probably caused apoptosis in these leukemia cells through the activation of caspase-3 and degradation of PARP [24]. From the culture filtrates of the same fungus *A. flavipes*, aspergilasines A-D (**15–18**, Fig. 3) four new merocytochalasans, were also isolated. Aspergilasines B-D (**16**–**18**) could be key biosynthetic intermediates of epicochalasine B [25].

From the solid culture of another strain of the same fungus, flavichalasines A and B (**19** and **20**, Fig. 3), flavichalasines C-E (**21**–**23**, Fig. 3), and flavichalasines F-M (**24**–**31**, Fig. 3), two tetracyclic, three pentacyclic, and eight tricyclic new cytochalasans, respectively, were isolated together with eight known analogues. The latter cytochalasins were identified as aspochalasin D, E, H, M, Q and T, trichalasin H, and aspergillin PZ **32-39**, Fig. 3). Flavichalasines were assayed for in vitro cytotoxicity against seven human cancer cell lines (Jurkat, HL60, NB4, 231, HEP-3B, HCT116, and RKO) using taxol as positive control. Flavichalasine F **24** and aspochalasin E (**33**) showed moderate cytotoxic activitiy, with IC_50_ values ranging from 9.6 to 26.6 μM. The other compounds had no significant toxicity effects. Furthermore, the same two cytochalasins **24** and **33**, induced significant apoptosis of HL60 cells compared to the control. Finally, the latter two compounds also altered the expression levels of caspase-3 suggesting that they caused apoptosis through activation of caspase-3 and degradation of PARP [26].

Working with the same fungus *A. flavipes* but with another strain QM507, collected in a differ China province, flavipesines A and B (**40** and **41** Fig. 3) and asperchalasines E–H (**42**–**45**, Fig. 3), two cytochalasans with an unusual ring system 5/6/7/6 ring system with a C-18−O−C-21 bridge. And four merocytochalasans possessing a 5/6/11/5/5/6 ring were isolated together with asperchalasines B-D (**7**–**9**). Compounds **42**, **7**, and **9** show moderate inhibitory activities against isocitrate dehydrogenase 1 (IDH1). This is the first report on the IDH1 inhibitory activities of cytochalasans. All cytochalasins were tested for their ability to inhibit isocitrate dehydrogenase 1 (IDH1) using the compound AGI-5198,15 as a selective inhibitor of the R132H mutantIDH1, as a positive control (IC_50_ of 0.2 μM). Compounds **42**, **7**, and **9** showed moderate inhibitory activity (IC_50_ values of 17.31, 11.66, and 13.31 μM, respectively). To further clarify the inhibitory mechanism on IDH1R132H, a molecular docking study was conducted, demonstrating that the compounds bind to an allosteric site, locking the enzyme in a catalytically inactive conformation. The ligand-binding pocket of DH1R132H has a croissant-dove shape with many hydrophobic amino acids (Trp124, Ile128, Ile130, His132, Ile251, Met254, Val255, Phe265, Val276, and Cys269) that formed a highly hydrophobic shell. Cytochalasin **42** established clear hydrophobic interactions with many of these amino acids, such as the side chain Ile128, Ile130, Phe265, Val276, and Val281, and hydrogen bonds between the phenolic hydroxy of **42** with Ser278 and Ser280 [27].

##### Cytochalasins isolated from *Aspergillus iizukae*

**Fig. 4:**
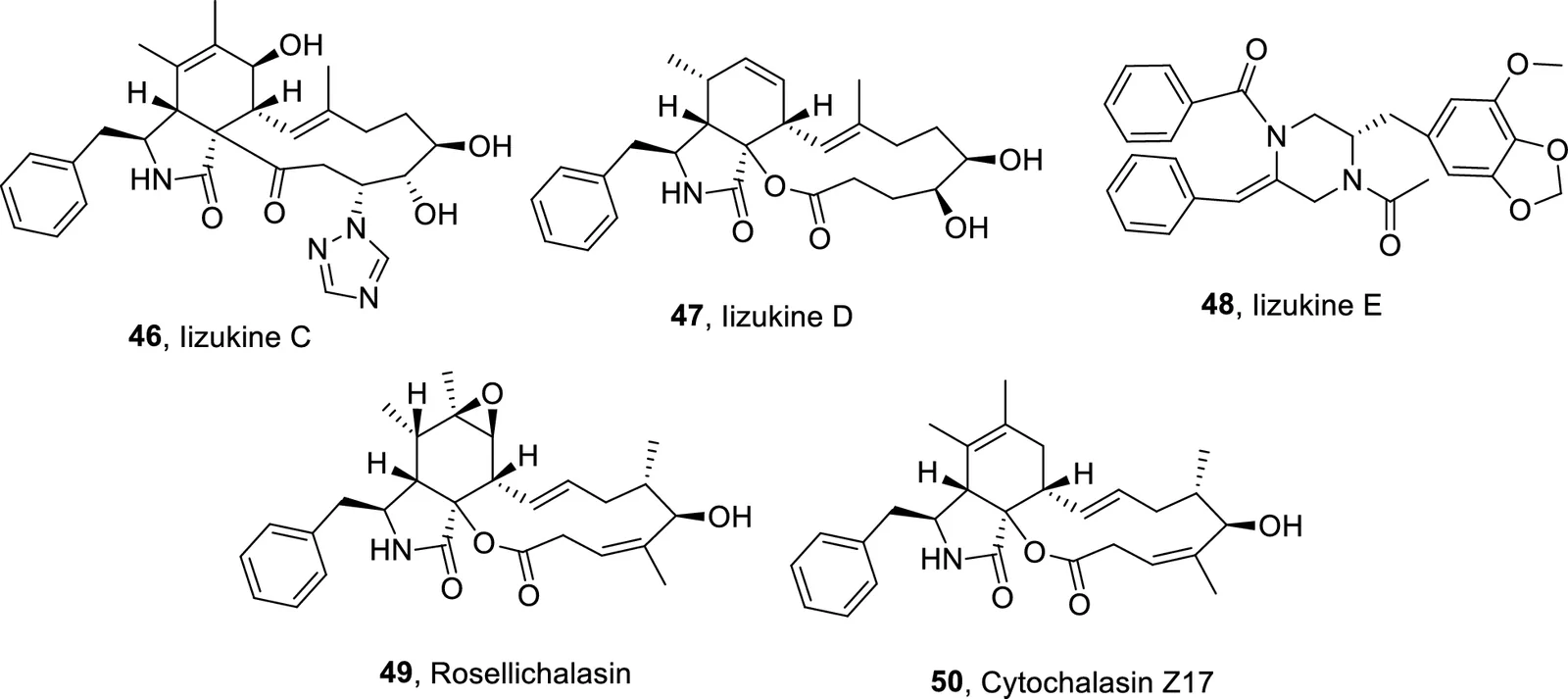
Cytochalasins isolated from *Aspergillus iizukae*

From another *Apergillus* species, namely *A. iizukae*, lizukines C-E (**46**–**48**, Fig. 4) including two aspochalasins and one brasiliamide derivatives were isolated, together with the already known rosellichalasin and cytochalasin Z17 (**49** and **50**, Fig. 4) Compound **45** was the first aspochalasin having a 1,2,4-triazole moiety joined to the macrocyclic ring. The three compounds **46**–**48** were tested for their cytotoxic activity against HL-60 and A549 human cell lines using aspochalasin E (36) as positive control (IC_50_ 1.8 and 3.1 μM respectively). Compounds **46** showed cytotoxic effect with IC_50_ values of 3.8 and 7.2 μM, respectively, whilst cytochalasins **47** and **48** were no toxic up to a concentration of 20 μM [28].

#### Cytochalasins isolated from *Xylaria* species

Fungi of the genus *Xylaria* are widespread from temperate to tropical zones of the Earth and are commonly found on stumps and fallen branches of deciduous trees. Most species are white-rot-inducing fungi and produce phytotoxic/bioactive metabolites, including cytochalasins [29].

##### *Cytochalasins isolated from Xylaria striata and Xylaria longipes*

**Fig. 5:**
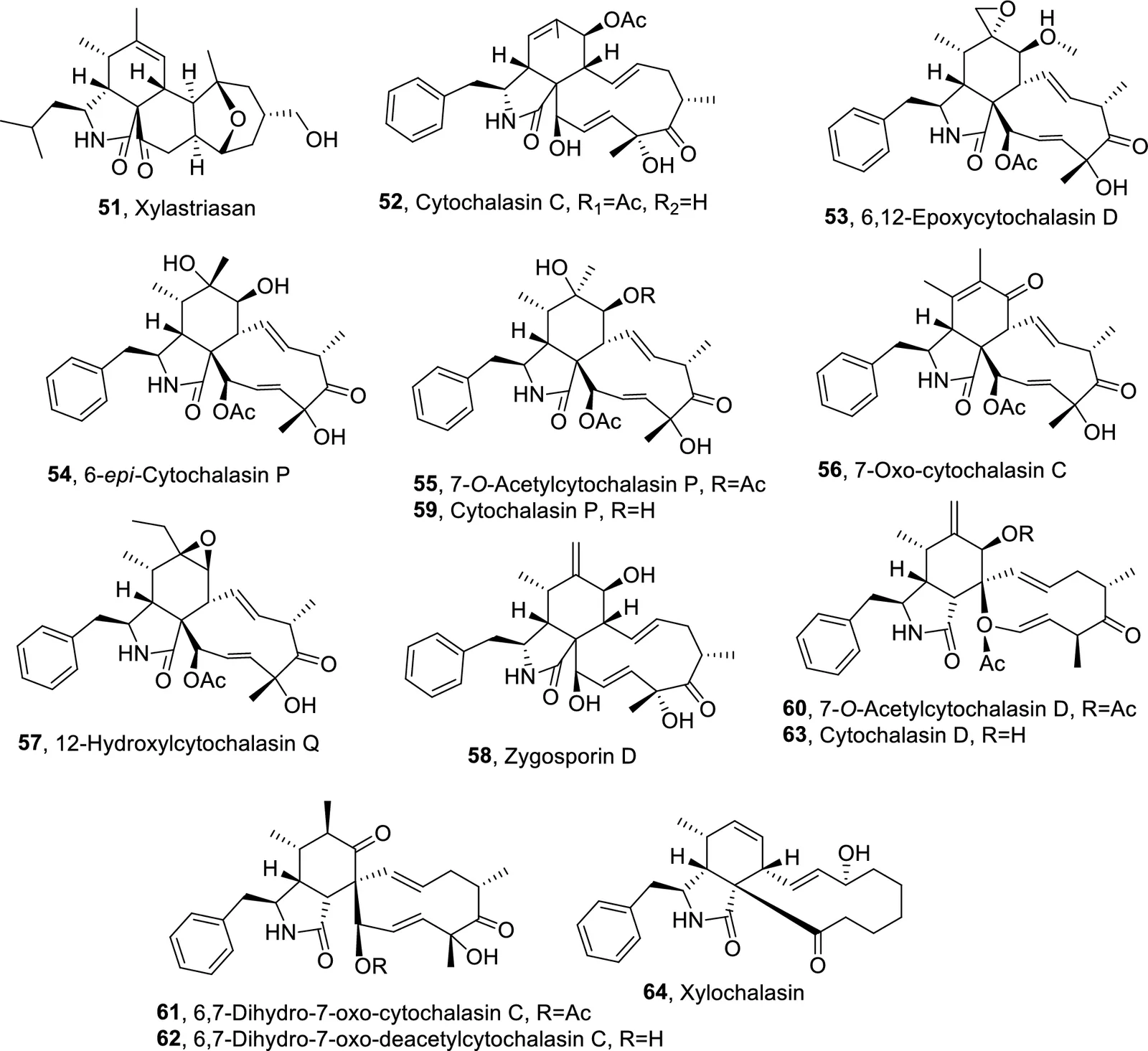
Cytochalasin isolated from *Xylaria striata*, *Xylaria longipes* and *Xylaria* sp. SWUF08-37

Xylastriasan A (**51**, Fig. 5) and ergosterol were isolated from the fruiting bodies of the fungus *Xylaria striata*. Compound **51** showed moderate cytotoxicity against HEPG2, B16 and A549 cell lines with IC_50_ values of 93.61, 85.61 and 91.58 μM, respectively. Ergosterol potentiated pentobarbital-induced sleep by not only increasing the number of falling asleep and prolonging sleeping time but also reducing sleep latency at a dosage of 5 mg/kg [30]. Later, from *Xylaria longipes* cytochalasin C, 6,12-epoxycytochalasin D, 6-*epi*-cytochalasin P, 7-*O*-acetylcytochalasin P, 7-oxo-cytochalasin C, 12-hydroxycytochalasin Q (**52**–**57**, Fig. 5) were isolated together with zygosporin D, cytochalasin P, 7-*O*-acetylcytochalasin D, 6,7-dihydro-7-oxo-cytochalasin, 6,7-dihydro-7-oxo-deacetylcytochalasin C and cytochalasin D (**58**–**63** Fig. 5), All cytochalasins were tested for their cytotoxicity against human cancer cell lines HL-60, A549, SMMC-7721, MCF-7, and SW480. Cytochalasins P and D, zigosporin D, 6,7-dihydro-7-oxo-cytochalasin C, and 6,7-dihydro-7-oxo-deacetylcytochalasin C showed cytotoxicity with IC_50_ value ranging from 4.17 to 37.18 μM. SAR investigation showed that the 7-OH and C-11 substituent are important features for the toxicity against HL-60 cell line [31].

##### Cytochalasin isolated from *Xylaria* sp. SWUF08-37

Xylochalasin (**64** Fig. 5), a new cytochalasin and pentaminolarin, a cyclic pentapeptide, were isolated together with thirteen known compounds, including pyrones, phenols and sterols, from the wood-decaying fungus *Xylaria* sp. SWUF08-37. The cytotoxic activity of almost all compounds (those isolated lesser than 10 mg were not tested) was assayed toward human carcinoma cell lines (Hela, HT29, HCT116 and MCF-7) and normal Vero cells. The results showed that xylochalasin (**64**) and pentaminolarin had slight cytotoxicity while the epoxycyclohexenone 4S,5S,6S-4-hydroxy-3-methoxy-5-methyl-5,6-epoxycyclohex-2-en-1-one showed toxicity against all the tested cell lines [32].

##### Cytochalasins from other fungi

**Fig. 6:**
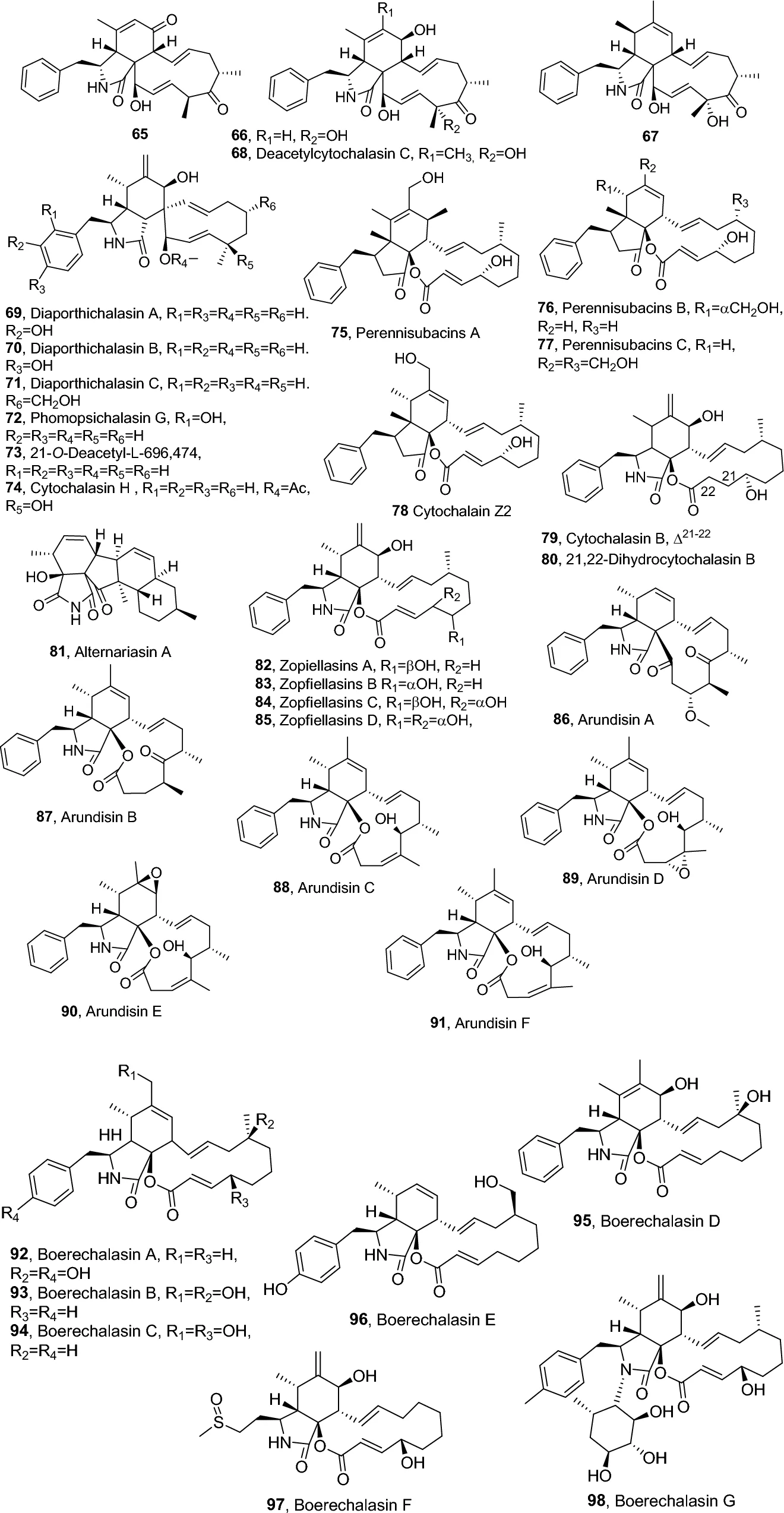
Cytochalasins isolated from other fungal genera

*Coryceps taii*, is a species of the genus *Cordyceps* discovered in Guizhou province of China in the 1990s. These fungi, which mainly parasitize the larvae of insects or/and arthropods, was used in folk Chinese medicine or as tonics for hundreds of years and had successively received increasing interest around the world. Indeed, *C. taii* showed significant pharmacological activities, such as immunomodulatory, anti-aging, antioxidant, and antimicrobial effects [33]. From the ethanol extract of this mushroom, which showed antitumor activity in vitro and in vivo, three new cytochalasins (**65**–**67**, Fig. 6) were isolated from together with zygosporin D and deacetylytochalasin C (**68**, Fig. 6). The cytochalasins **63**–**65** were tested in vitro against human highly metastatic lung cancer cell 95-D, human lung cancer cell line A-549 and normal hepatocyte HL-7702. Compounds **63** and **64** showed potent antitumor activities against human lung cancer cell 95-D with IC_50_ value of 3.67 and 4.04 **μ**M, respectively. They had little or no cytotoxicity on normal cells, but showed strong cytotoxic effects on cancer cells 95-D when compared with cisplatin used as positive control [34].

The genus *Diaporthe* (sexual form of *Phomopsis*) includes many plant pathogens that cause various diseases in host plants, such as cankers, blights, root rots, fruit rots and leaf spots [35] Previous chemical studies have shown that *Diaporthe* or *Phomopsis* species could produce a variety of bioactive metabolites, such as cytotoxic sesquiterpenoids, cytotoxic xanthone dimers and azaphyllones, antibacterial triterpenoids and anti-inflammatory cytochalasins [36]. From the fungal solid culture organic extract of *Diaporthe* sp. GZU-1021D diaporthichalasins A-C (**69**–**71**, Fig. 6) were isolated together with the already known phomopsichalasin G, 21-*O*-deacetyl-l-696,474 and cytochalasin H (**72**–**74**, Fig. 6), biatriosporin N, phomopsichin B, penialidin A and phomoxanthone A from the culture of the fungus. All compounds were tested for their inhibitory activities against LPS-activated NO production in RAW 264.7 cells, using indomethacin as positive control (IC_50_ 29.7 μM). 21-*O*-Deacetyl-l-696,474, cytochalasin H, biatriosporin N, phomopsichin B, penialidin A and phomoxanthone A inhibited NO production with IC_50_ values ranging from 1.94 to 16.5 μM. The results showed that diaporthichalasins A-C and phomopsichalasin G, 21-*O*-deacetyl-l-696,474 and cytochalasin H, which shared the same cytochalasin skeleton, had different inhibitory activity and this is interesting in the SAR point of view. In fact, the monosubstituted benzene ring could play a role in the antiinflammatory activity as resulted by comparison of the structures of diaporthichalasins B (no inhibitory activity) and 21-*O*-deacetyl-l-696,474 (IC_50_ 7.35 mM). The hydroxyl and the acetyl group at C-18 and C-21, respectively, present in cytochalasin H (IC_50_, 1.94 μM; CC_50_ (50% cytotoxic concentration), 3.84 μM) should increase the bioactivity and cytotoxicity as resulted by comparison to those of 21-*O*-deacetyl-l-696,474 [36].

Perennisubacins A-C (**75**–**77**, Fig. 6), together with the already known cytochalasin Z2, cytochalasin B and its 21,22-dihydroderivative (**78–80**, Fig. 6) were isolated from the fungus *Perenniporia subacida*, which is one of the most common and widespread polypore in Asia, Europe, and North America [37]. Cytochalasins **75** and **77** showed cytotoxicity against SMMC-7721 and A-549 cell lines with IC_50_ value of 23.3 and 37.6 μM, respectively. Furthermore, compound **75** inhibited nitric oxide (NO) production with an IC_50_ value of 18.4 μM [38].

*Alternaria alternata* belong to a special ecological niches, which appeared interessting for their ability to produce different bioactive secondary metabolites [39] Alternariasin A (**81**, Fig. 6), a new cytochalasin, was isolated together with altertoxin I, altertoxin II, tenuazonic acid, aloesol and alternariol from the solid cultures of the Midui Glacier-derived fungus. All the compounds were tested for antibacterial activity against *S. aureus*, *B. subtilis*, *S. pneumoniae* and *E. coli*. Cytochalasin **81** showed toxicity against *S. aureus*, *B. subtilis* and *E. coli* with MIC values of 16.7, 16.7 and 33.3 μΜ, respectively, while ampicillin and gentamicin, used as positive controls, showed MIC values of 0.06, 1.28 and 2.09 μΜ, respectively. The other compounds were not toxic even at 80 μM. All the compounds were also assayed for cytotoxic activity toward five human cancer cell lines, Hela, HepG2, A549, MCF-7, HCT116 and one human glioma cell lines SHSY5Y. Only altertoxin II showed cytotoxicity against all the cell lines tested, showing IC_50_ values of 6.3, 7.6, 12.7, 17.6, 5.4 and 6.3 µM, respectively, while cisplatin, used as positive control, exhibited IC_50_ values of 14.1, 15.8, 17.9, 18.2, 9.5 and 12.7 µM [40].

Zopfiellasins A-D (**82**–**85**, Fig. 6), two new pairs of epimeric cytochalasins, were isolated from *Zopfiella* sp. a fungus associated to kiwi and collected during a screening aimed to find anti *Pseudomonas syringae* pv. *actinidiae* (*Psa*). The latter is the most devastating and distructive pathogen of kiwi orchard [41]. Only zopfiellasins A and C (**82** and **84**) inhibited *Psa* (MIC (Minimum Inhibitory Concentration) values of 25 and 50 g/mL, respectively). These results suggested that the configuration of C-19 might play an important role in the antibacterial ability. In fact, the opposite *S* stereochemistry of C-19 in compounds **83** and **85** determined the lacking of any toxicity [42].

The fungus *Arthrinium arundinis*, obtained from the root of *Aconitum brevicalcaratum* (Ranunculaceae), was known to produce cytochalasans and alkaloids such as 4-hydroxy-2-pyridone alkaloids [43]**.** In fact, arundisins A-F (**86**–**91**, Fig. 6), four new cytochalasins, were isolated together with to several known cytochalasins from the organic extract of fungal solid culture. Arthriniumnins A-D, as well as five known cytochalasin analogues were also isolated from the same fungus but when it was collected from the sponge *Phakellia fusca* as reported in the section “2.3 Cytochalasins from marine organism”. Cytochalsins **86**–**91** were tested for their cytotoxic activity against five cell lines, inhibitory effect on nitric oxide production in LPS-activated RAW264.7 macrophages and antimicrobial activity against *S. aureus*, *B. subtilis*, *C. albicans* and *E. coli*. Only compounds **86**–**88** showed cytotoxic activities against five cell lines, and were more cytotoxic to MCF-7 than the positive control (cisplatin, IC_50_ 23.96 ± 1.02 μM). Furthermore, arundisins F (**91**) had toxicity towards against *E. coli* (MIC 8.00 μg/mL) while compound **87** showed moderate antifungal activity against *C. albicans* (MIC of 32.00 μg/mL). All the cytochalasins had no inhibitory effects on nitric oxide production in LPS-activated RAW264.7 macrophages [44].

Boerechalasins A-G (**92**–**98**, Fig. 6) were isolated, together with cytochalasin B from the solid culture of the fungus *Boeremia exigua*, which caused different diseases on various plants such as *Luobuma* plants and *Morus malba* in China, *Fraxinus excelsior* in Poland, *Hydrangea paniculate* in Italy, *Cichorium intybus* var. *sativum* in Chile and *Cycas circinalis* in India [45]. An unusual sulfoxide moiety, which might be derived from methionine, and an unusual 5-methylcyclohexane-1,2,3-triol substituent at N-2 position, were present in both boerechalasins F and G (**97** and **98**), respectively. Cytochalasins **92**–**98** were tested for their cytotoxic activity and compound **97** showed cytotoxicity against MCF-7 (IC_50_ value of 22.8 μM) while compounds **92** and **96** exhibited anti-inflammatory activity (IC_50_ values of 21.9, and 5.7 μM, respectively). These results suggested that the OH group at C-4′, present in both cytochalsins could cause their inability to inhibit NO production [46].

### Cytochalasins from endophytic fungi

Endophytic fungi, like bacteria, live essentially in all plant species, but not exclusively, without causing harmful effects to their hosts. They promote plant growth by improving nutrient exchange and enzymatic activity. Therefore, they have a significant impact on the physiological processes that take place within their hosts by synthesizing a wide range of secondary metabolites. These metabolites, that include hundreds cytochalasins, often exhibit diverse biological activities and potential applications in diverse fields such as agriculture and medicine [15, 16, 47, 48]

#### Cytochalasin from *Trichoderma* spp.

The genus *Trichoderma* includes fungi capable of colonizing a wide variety of substrates under diverse environmental conditions. They can survive as saprophytes on plant debris and, in some cases, colonize living plant tissue. This ability is due in part to their ability to produce various secondary metabolites, including antimicrobial compounds that can enhance their antagonistic activity as biocontrol agents for crop diseases caused by other fungi [49].

**Fig. 7:**
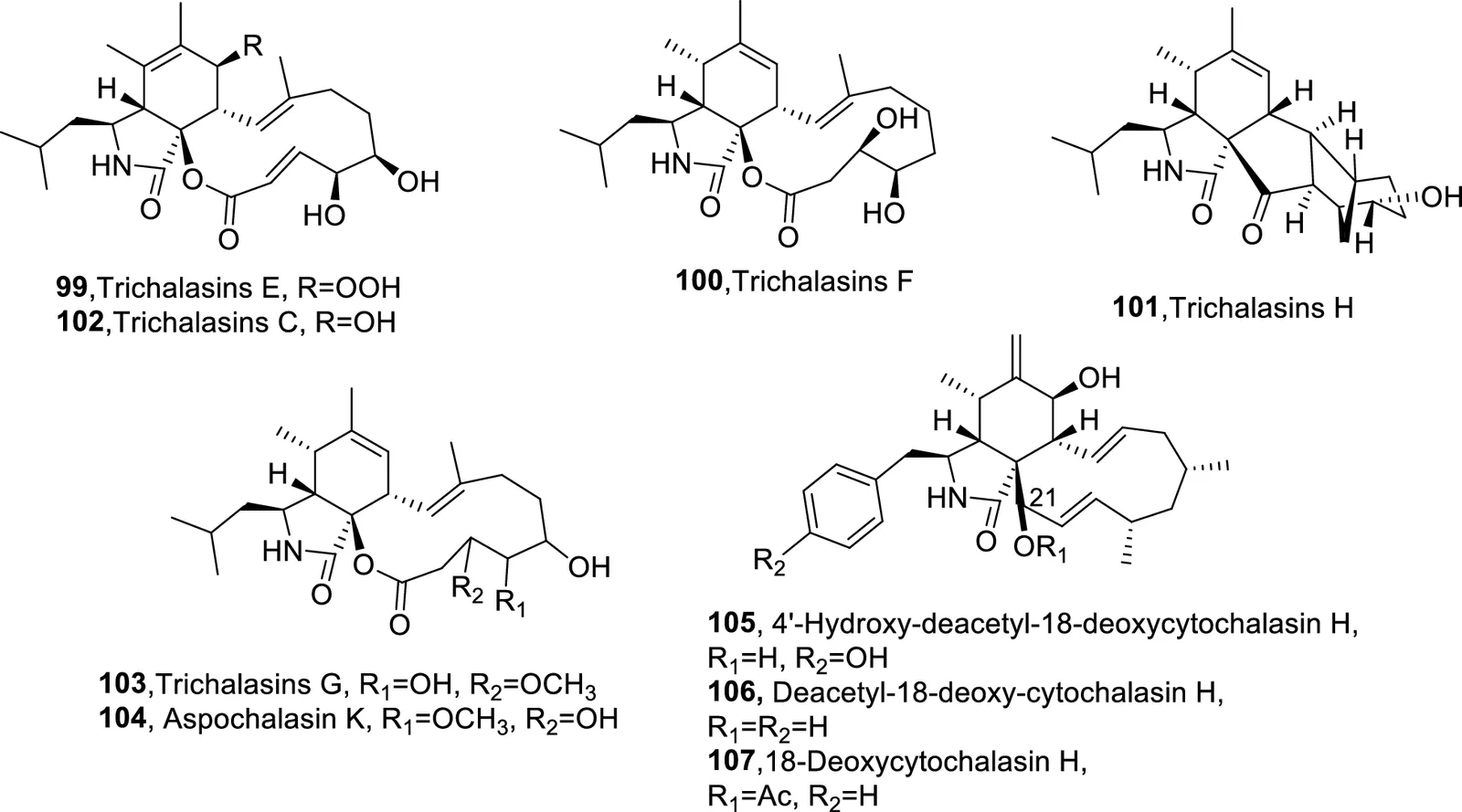
Cyochalasins isolated from *Trichoderma* species

From the endophytic fungus *Trichoderma gamsii*. collected from *Panax notoginseng*, a plant used in Chinese folk medicine, trichalasins E, F and H (**99**–**101**, Fig. 7), three new cytochalasins, were isolated together with the already known analogues trichalasins C and G, and aspochalasin K (**102** and **103** and **104**, Fig. 7). Trichalasins E (**99**) contains a unique hydroperoxyl group and this is the first report among all known analogues. All compounds were tested for their inhibitor activity toward the A549, MDA-MB-231 and PANC-1 cancer cell lines and trichalasin G (**103**) and aspochalasin K (**104**) showed moderate activity against MDA-MB-231 cell line (IC_50_ value of 60.6 μM) [50].

4'-Hydroxy-deacetyl-18-deoxycytochalasin H, deacetyl-18-deoxycytochalasin H 18-deoxycytochalasin H (**105**–**107**, Fig. 7) were isolated from the endophytic fungus *Trichoderma harzianum*, which was obtained from leaves of *Cola nitida*. This plant as other *Cola* species was used for preparation of caffeine. All compounds isolated were tested for their cytotoxicity against L5178Y, A2780 sens, and A2780 CisR cancer cell lines. Cytochalasin **107** showed the strongest activity against all the three cancer cells with IC_50_ values of 0.19, 0.42, and 6.55 μΜ, respectively. In the SAR point of view the deacetylation of the hydroxy group at C-21, as occurred in compounds **105** and **106**, caused a significant decrease of the activity, suggesting that the acetate group plays an important role to impart cytotoxicity. The well known lethal metabolisms could explain this results as the acetate group enhanced the membrane permeation and it could be then hydrolized. Furthermore, the presence of an additional hydroxyl in the aromatic ring of **105** affect the activity of this compound [51].

#### Cytochalasins isolated from *Xylaria* spp.

*Xylaria* species are world spread fungi as above cited as well as also as endophytes of different organisms.

**Fig. 8:**
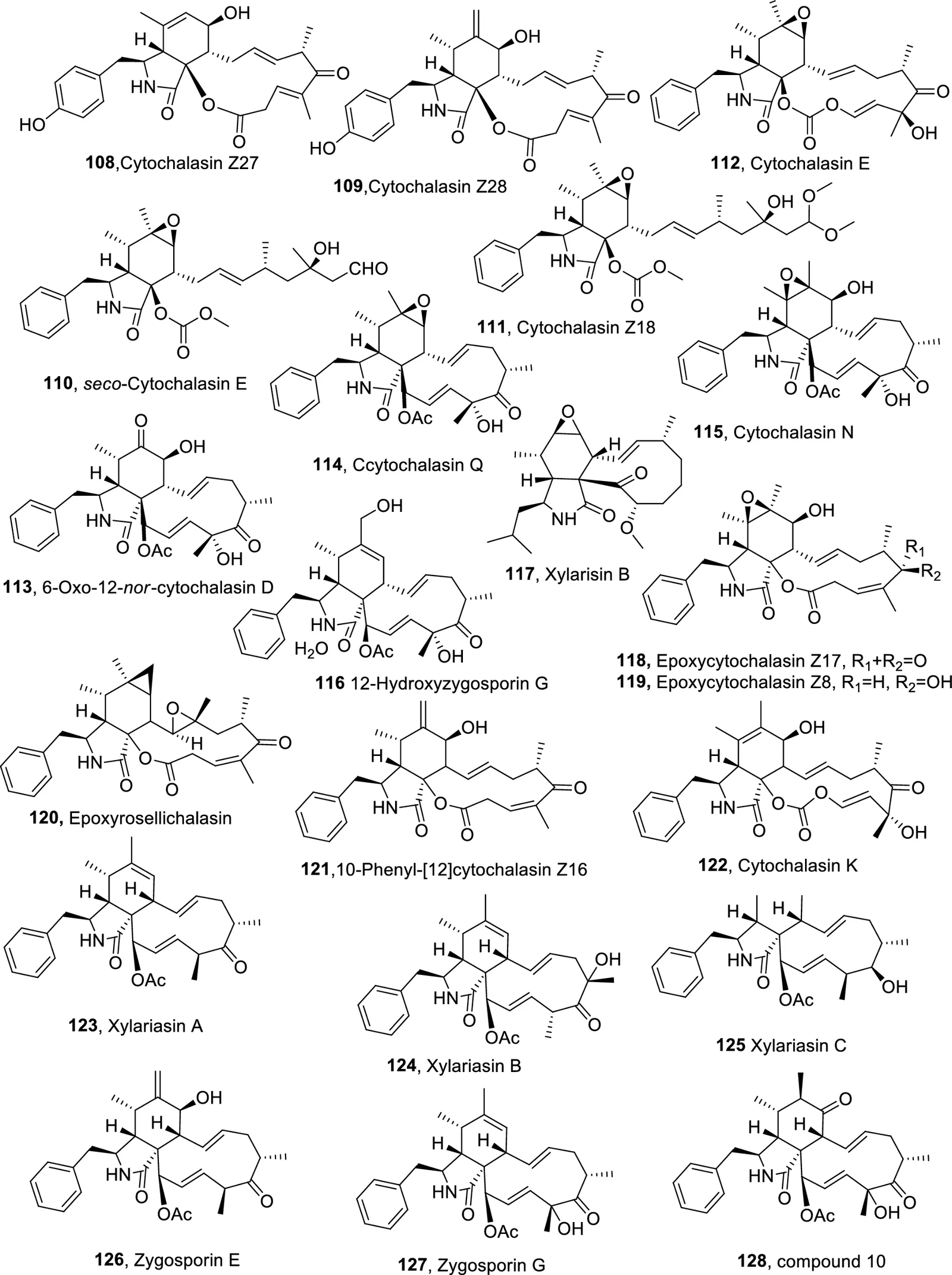

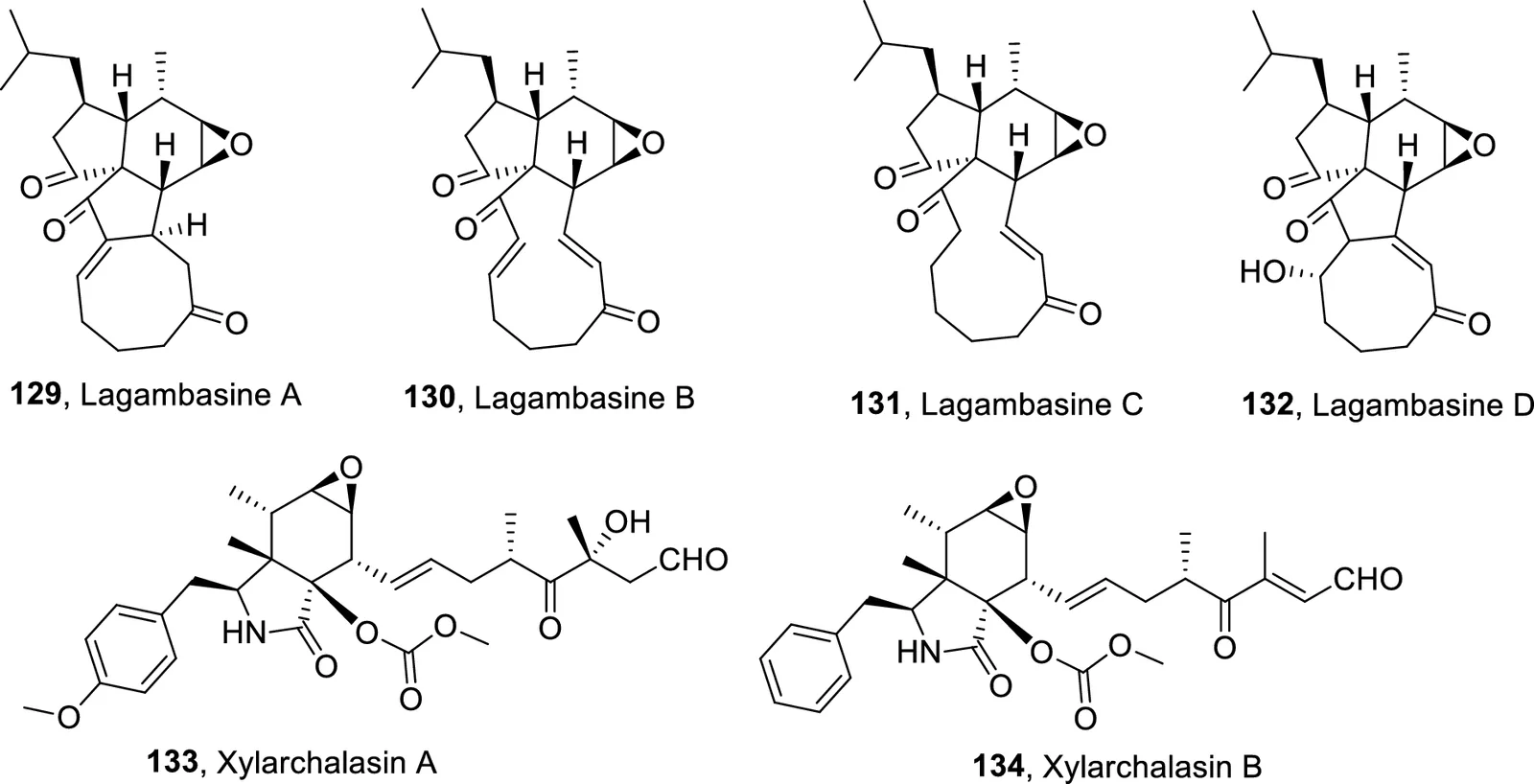
Cytochalasin isolated from endophyte *Xylaria* spp.

From *Xylaria* sp., an endophyte fungus collected from *Toona sinensis*, which is a plant known as Chinese toon and belonging to the family of the Meliaceae used in popular medicine for its anticancer and anti-inflammatory effects, were isolated Cytochalasin Z27 and cytochalasin Z28, (**108** and **109**, Fig. 8). From the same fungus the already known seco-cytochalasin E, cytochalasin Z18, and cytochalasin E, (**110**–**112**, Fig. 8) were isolated too [52]. Cytochalasin E (**112**) showed strong cytotoxic activity against brine shrimp (LC_50_ 2.79 μM), comparable to that of the positive control toosendanin (LC_50_ 4.03 μM). The same cytochalasin, at concentrations in the range of 10–80 μM, also exhibited higher phytotoxic effects on *Lactuca sativa* and *Raphanus sativus* L. seedlings than that of the positive control glyphosate, which is a broad-spectrum systemic herbicide in agriculture. On the contrary, all the other compounds have not shown any effect on both seedlings even at concentration of 100 μM. These results showed again that a carbonate vinyl of the 13 members macrocyclic ring could be important in determining phytotoxic activity. In fact, the opening of macrocycle as happens in the conversion of cytochalasin E (**112**) in its seco-analogue **110** leads to the loss of activity. Furthermore, cytochalasin Z28 (**109**) showed higher fungicidal activity towards *Giberella saubinetti* than that of hymexazol used as positive control [53].

From another *Xylariaceae* sp. strain, a fungal endophyte inhabiting the Spanish moss, *Tillandsia usneoides*, were isolated 6-oxo-12-*nor*-cytochalasins D, cytochalasins Q and N, and 12-hydroxyzygosporin G (**113**–**116**, Fig. 8) together with the well known cytochalasins D, 4,5-di-isobutyl-2(1H)-pyrimidinone, heptelidic acid chlorohydrin, (+)-heptelidic acid, and trichoderonic acid A. All cytochalasin were tested for their anti-cancer activity against a panel of five human tumor cell lines (non-small cell lung (NCI-H460), CNS glioma (SF-268), breast (McF-7), metastatic breast adenocarcinoma (MDA-MI-B-231), and metastatic prostate adenocarcinoma (PC-3 M) cancer cell lines, using doxorubicin and DMSO positive and negative controls, respectively. Among all the cytochalasins tested, cytochalasin D showed the strongest cytotoxicity on all cell lines with IC_50_ of 0.2–1.4 μM and a certain degree of selectivity on the NCI-H460 cell line with respect to four other cell lines. Cytochalasin Q (**114**) also showed cytotoxicity against all cell lines (IC_50_ 1.3–1.5 μM) except on MCF-7, while cytochalasin N (**119**) and 6-oxo-12-norcychalasin D (**113**) had a moderate selective cytotoxic activity on SF-268 (IC_50_ 2.9 μM) and PC-3 M (IC_50_ 3.8 μM) [54].

Xylarisin B (**117**, Fig. 8) was isolated together with four known compounds, namely astropyrone, guaidiol, xylaropyrone B, and xylaropyrone C from *Xylaria* sp. HNWSW-2, which is an endophyte fungus collected from the stems of *Xylocarpus granatum*. All the compounds were assayed for their AChE activity at a concentration of 50 μg/mL, using tacrine as positive control and showing an inhibition rate of 77.4%. Astropyrone and guaidiol exhibited a moderate inhibitory activity against AChE with inhibition rates of 10.4 and 12.9%, respectively. When assayed at a concentration of 0.25 mg/mL and using acarbose as positive control (inhibition rate of 59.7%) astropyrone also showed α-glycosidase inhibition rate of 77.0% [55].

Epoxycytochalasin Z17, epoxycytochalasin Z8, and epoxyrosellichalasin, (**118**–**120**, Fig. 8) were isolate together with cytochalasins E and Z17 and 10-phenyl-[12]-cytochalasins Z16 and cytochalasin K (**121** and **122**, Fig. 8) from the culture of the endophytic fugus *Xylaria* sp. XC-16 inhabiting in a deciduous tree *Toona sinensis*. Hydroxyldecandrin G, an abietane-type diterpenoid, with four known analogues were also isolated from the same fungal cultures [56]. Among the isolated compounds, many showed allelopathic activity against two herbaceous plants, turnip (*Raphanus sativus*) and wheat (*Triticum aestivum*). In particular, cytochalains **120** and **122** and hydroxyldecandrin G, strongly inhibited wheat shoot elongation, and 10-phenyl[12]cytochalasins Z16, K and E inhibited wheat root elongation, showing comparable IC_50_ values to that of the positive control glyphosate. Cytochalasin E also was a potential inhibitor on turnip root elongation (IC_50_ value of 1.57 ± 0.21 μM), a value which was 50-fold more potent than glyphosate. However, xylarisin B and cytochalsin K stimulated turnip shoot elongation at lower concentrations [56].

**Fig. 9:**
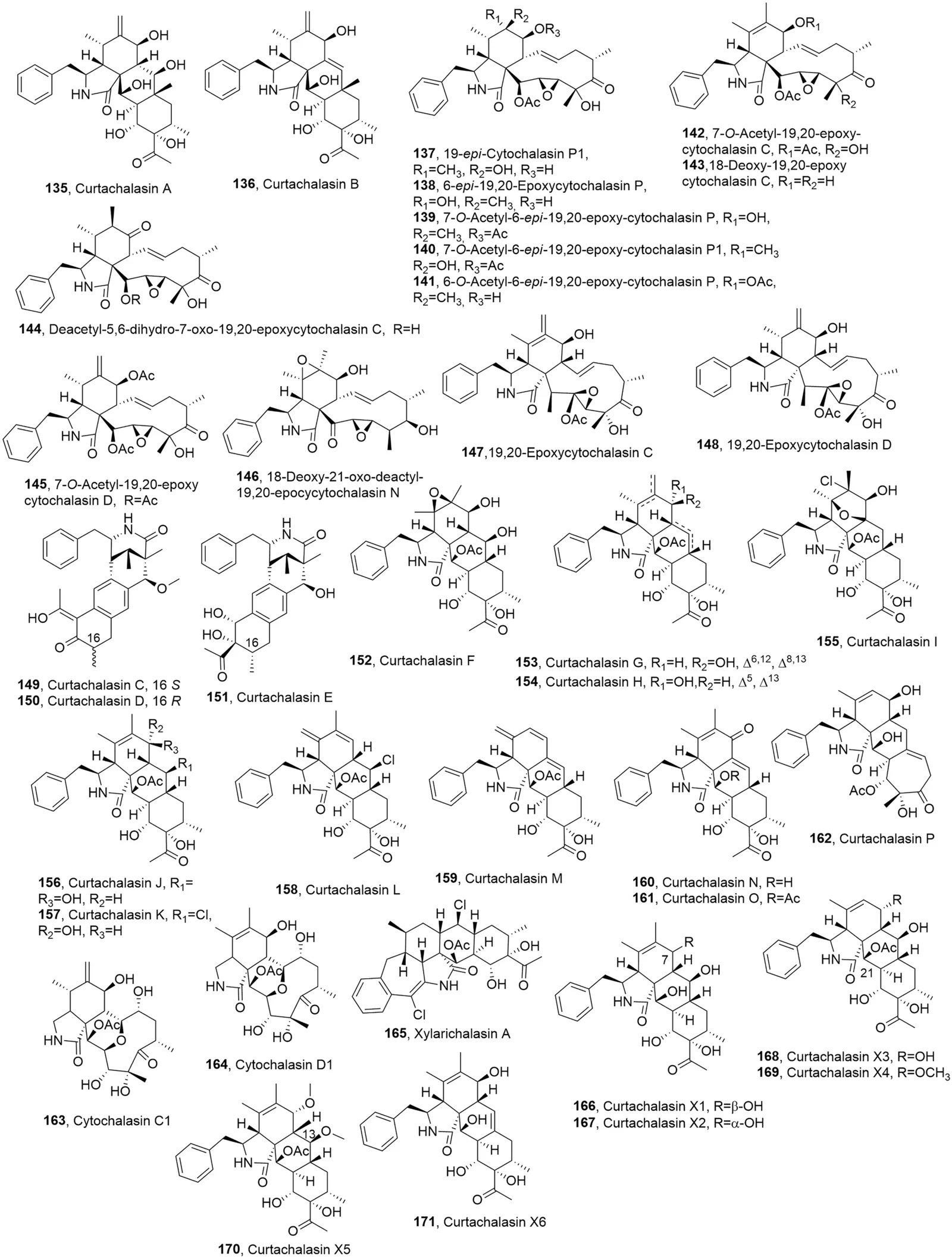
Cytochalasins isolated from the endophyte *Xylaria curta*

From the strain *Xylaria* sp. CFL5, an endophytic fungus of *Cephalotaxus fortune*, xylariasins A-C (**123**–**125**, Fig. 8), three new cytochalasins, were isolated together with cytochalasins C and D and 12-hydroxylzygosporin G, and zygosporin E, zygosporin G and compound 10 (**126**–**128** Fig. 8). The cytotoxic activity of all cytochalasins was tested through their effect on LAG3/MHC II binding inhibition and LAG3/FGL1 binding inhibition activities. Cytochalasins C and D showed cytotoxicity toward AGS cells at 5 μM, with inhibition rates of 94% and 64%, respectively. In addition, all compounds, except cytochalasin D, showed activity against the interaction of both LAG3/MHC II and LAG3/FGL1. Xylariasin A (**123**), 12-hydroxylzygosporin G, zygosporin E (**126**) and compound 10 (**128)** inhibited LAG3/MHC II (IC50 values ranging from 2.37 to 4.74 μM), while the IC_50_ values of xylariasin A, 12-hydroxylzygosporin G and zygosporin E against LAG3/FGL1 were 11.78, 4.39, and 7.45 μM, respectively [57].

Lagambasines A-D (**129–132** Fig. 8), four new cytochalasins, were isolated from a cultivated endophytic fungus *Xylaria* sp. strain WH2D4 (Xylariaceae), which was collected from the leaves of the neotropical tree species *Palicourea elata* (Sw.) Borhidi (Rubiaceae) in Costa Rica. Cytochalasins **129–132** were assayed at a concentration between 10, 20, 30 and 80 **μ**g/disc in a disk diffusion assay. Any compound inhibit the opportunistic fungi species such as *Trichoderma reesei* QM6a and *Trichoderma longibrachiatum* UAMH 7956 confirming the resistance of T*richoderma* species against potentially antifungal agents [58].

From the endophytic fungus *Xylaria* sp. GDGJ-77B, collected from the Chinese medicinal plant *Sophora tonkinensis*, xylarchalasins A and B (**133** and **134**, Fig. 8) were isolated together cytochalasins Z17, Z18, E and K, asperchalasin D and cytochalasin Z27. All the compounds were tested against Gram-positive bacteria *B. anthracis*, *B. megaterium*, *B.* *subtilis, Bacillus paratyphosus,* and *S. aureus*, and Gram-negative bacteria *E. coli* and *Shigella dysenteriae*. Xylarchalasins A (**133**), asperchalasin D, cytochalasin Z18 and cytochalasin K exhibited antibacterial activities against. *B. megaterium*, *B. anthracis*, *E. coli* and *B. subtilis* (MIC value of 25**μ**g/mL. Compounds **133** and cytochalasin E showed antibacterial activities against *B. su*btilis and *E. coli* (MIC values of 25 and 12.5**μ**g/mL, respectively) and *B. anthracis* and *S. aureus* (MIC values of 25 and 12.5**μ**g/mL, respectively) [59].

#### Cytochalasins isolated from *Xylaria curta*

From the endophytic fungus *Xylaria curta* E10 collected from the plant *Solanum tuberosum* were isolated curtachalasins A and B (**135** and **136**, Fig. 9). These two cytochalasins were assayed for their cytotoxic activitiy towards five human cancer cell lines (HL-60, A-549, SMMC-7721, MCF-7, and SW480), for antibacterial activity against *E. coli*, *S. aureus*, *Salmonella enterica*, and *Pseudomonas aeruginosa*, and antifungal activity against *C. albicans*, *Epidermophyton floccosum*, *Trichophyton rubrum*, and *Microsporum gypseum*. Both showed moderate antifungal activity against *M. gypseum* (70.3 ± 0.4% and 68.4 ± 0.7% inhibitory percentage, respectively, at concentration of 200 μM) [60]. From the rice fermentation extract of another strain of the same fungus (*X. curta*), always obtained from potato (*S. tuberosum*), were isolated 19-epi, 6-epi-19,20-epoxy, and 7-*O*-acetyl-19-epi-cytochalasin P1, 6-epi-19,20-epoxy- and 7-*O*-acetyl-6-epi-19,20-epoxy- and 6-*O*-acetyl-6-epi-19,20-epoxy-cytochalasin P (**137**–**141**, Fig. 9) as well as 7-*O*-Acetyl-19,20-epoxy- 18-deoxy-19,20-epoxy- deacetyl-5,6-dihydro-7-oxo-19,20-epoxy-cytochalasin C, 7-*O*-acetyl-19,20-epoxycytochalasin D, and 18-deoxy-21-oxo-deacetyl-19,20-epoxycytochalasin N (**142**–**146**, Fig. 9) 19,20-Epoxycytochalasins C and D (**147** and **148**, Fig. 9) were also isolated from the same extract. The cytotoxic activity of all the cytochalasins was assayed against human cancer HL-60, A549, SMMC-7721, MCF-7, and SW480 cell lines. Noteworthy, only compound 19,20-epoxycytochalasin C (**145**) showed significant specific cytotoxicity against HL-60 cell lines with IC_50_ value of 1.11 μM [61]. Successively, curtachalasins C-E, (**149**–**151**, Fig. 9) were isolated too. A dose-dependent resistant reversal activity was observed when a combination of curtachalasin C (**149**) with 10 μg/mL of fluconazole were used against fluconazole resistance strains of *C. albicans*. Fluconazole is a commonly used drug for *C. albicans* infection, owing to its efficacy and low toxicity. The inhibitory activity reach 50%, by treatment with 16 μg/mL of cytochalasin **149** combined with 10 μg/mL of fluconazole, with a significant improvement compared to that of fluconazole alone. Furthermore, compound **149** alone had no inhibitory activity even at a concentration of 128 μg/mL. Curtachalasin E (1**51**) exhibited only moderate resistance reversal activity at a concentration of 128 μg/mL, while curtachalasin **150** was not assayed because of its low amount available [62]. Successively, curtachalasins F-P, (**152**–**162**, Fig. 9) were isolated from the same endophytic fungus and assayed for their cytotoxicity against splenocytes isolated from male BALB/c mice, together with curtichalasins A and B and D and E. Curtachalasins **151**, **154**–**156** and **158**–**160** and **162** did not show toxicity. On the contrary, curtachalasin F (**152**) exhibited a significant selective inhibition on B-cell proliferation (IC_50_ value of 2.42 μM) when tested in the immuno suppressive assay against concanavalin A (ConA) induced T lymphocyte cell proliferation and lipopolysaccharide (LPS) induced B lymphocyte cell proliferation. In the same test curtachalasin O (**161**) possessed selective inhibition on T-cell proliferation (IC_50_ value of 12.15 μM) [63]. Finally, also cytochalasins C1 and D1 (**163** and **164** Fig. 9) were isolated from *Xylaria* cf. *curta*. They were assayed for cytotoxicity against five human cancer cell lines, namely leukemia (HL-60), adenocarcinomic alveolar basal epithelial (A549), hepatocellular carcinoma (SMMC-7721), adenocarcinomic alveolar basal epithelial (A549), hepatocellular carcinoma (SMMC-7721), breast cancer (MCF-7), and colon adenocarcinoma (SW480) cell lines. Both cytochalasins **163** and **164** exhibited moderate cytotoxic activity only against HL-60 cell line, and compound **163** only showed slight activity against SMMC-7721 cell line [64]. Successively from the same fungus, xylarichalasin A (**165**, Fig. 9) was isolated and chemical characterized showing an unprecedented 6/7/5/6/6/6 fused polycyclic. Xylarichalasin showed significant cytotoxicity against SMMC-7721 and MCF-7 cell lines, stronger than the positive drug cisplatin. However, it showed only moderate activity against cell lines HL-60, A549, and SW480 [65]. Curtachalasins X1–X6 **166**–**171** Fig. 9) were isolated together with curtachalasins H–O (**154**-**161**) from the above cited endophytic fungus *Xylaria curta* E10. All cytochalasins were tested for their cytotoxic activity and using cisplatin as positive control. Curtachalasins **162** and **166** showed a very strong inhibitory activities (IC_50_ values of 2.03 and 0.85 μM, respectively) which were more potent than the positive control, cisplatin (IC_50_ 9.12 μM). In the SAR point of view the configuration of the hydroxyl group at the C-7 position, as was in compound 1**66** and **167**, may both important for the cytotoxicity. Furthermore, the methoxyl group at C-13 could be a key factor for toxicity as resulted comparing the activity of **168** and **170**. Also the acetylation of HO-21 appeared to be essential for the cytotoxicity as resulted comparing the activity of compound **167** with that of compound **168**. This latter result was in agreement with the cytotoxic activities previously observed with other cytochalasin derivatives [66].

#### Cytochalasins produced by *Xylaria arbuscola*

**Fig. 10:**
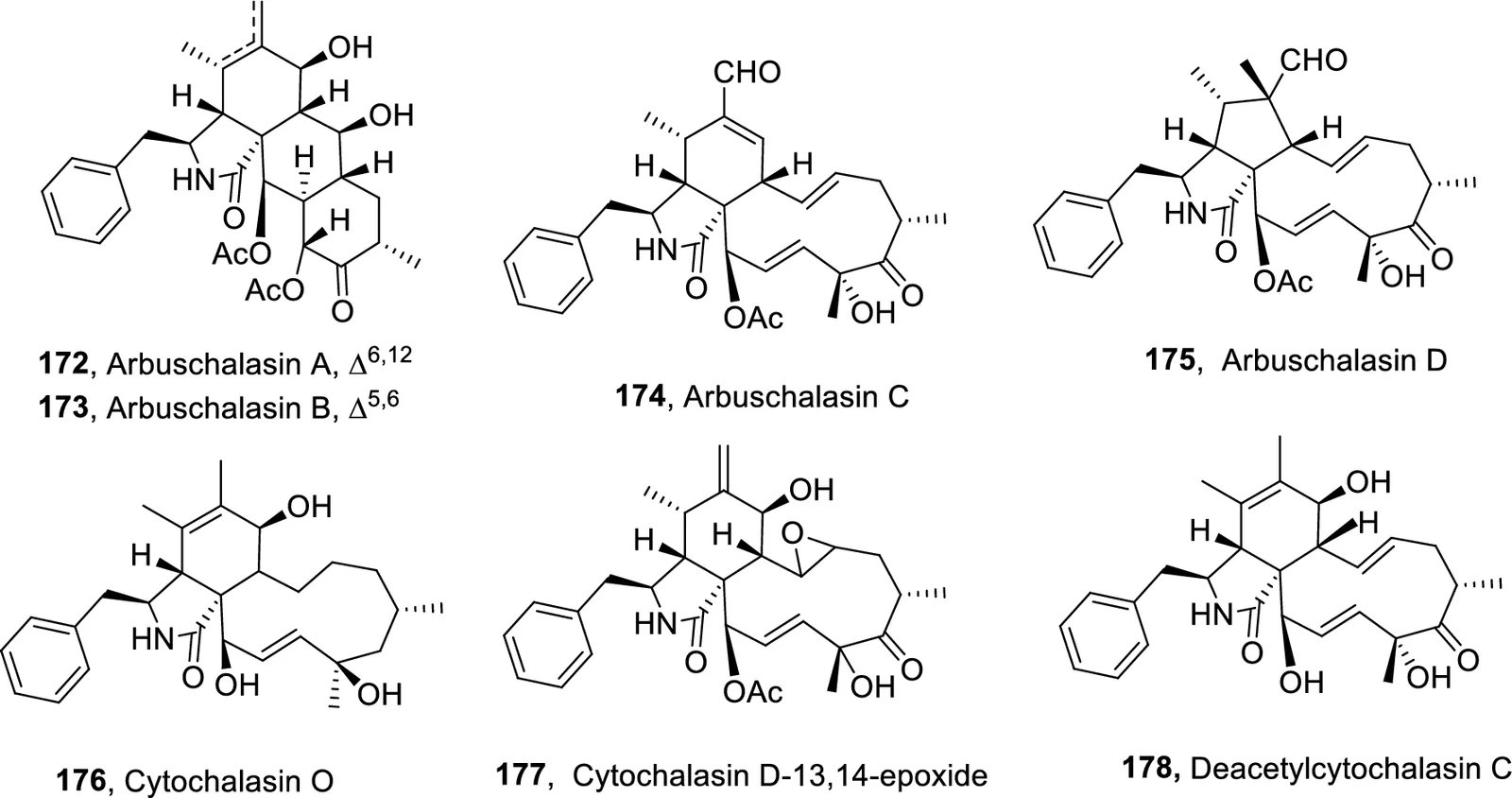
Cytochalasins from the endophyte *Xylaria arbuscola*

From the rice culture of endophytic fungus *Xylaria arbuscula*, collected from the mangrove plant *Bruguiera gymnorrhiza*, arbuschalasins A-D (**172**–**175**, Fig. 10) and cytochalasin O, 13,14-epoxide and deacetylcytochalasin C (**176**–**178**, Fig. 10) were isolated along with as cytochalasin Q, 12-hydroxylcytochalasin Q, zygosporin D, cytochalasin D, 19,20-epoxycytochalasin D, cytochalasin P, cytochalasin C, 6,7-dihydro-7-oxo-cytochalasin C, 6,7-dihydro-7-oxo-deacetylcytochalasin C, and 19, 20-epoxycytochalasin C (17) [67]. All metabolites were assayed for their cytotoxic activity against the human colorectal adenocarcinoma cell lines (HCT15). 12-Hydroxylcytochalasin Q and zigosporin D showed significant inhibitory activity (IC50 values were 13.5 and 13.4 μM, respectively), being more active than fluorouracil (103.1 μM) used as positive control. [67]. Arbuschalasins A-B (172–174) exhibit a rare 5/6/6/6 fused ring system, while arbuschalasin D is the first cytochalasin to possess a 5/5/11 fused scaffold. Arbuschalasins A-D (**172-174**), compared to common cytochalasins possessing the 5/6/11 fused scaffold, did not show significant inhibition of human colorectal adenocarcinoma cell lines (HCT-15). This finding suggests that the presence of the 5/6/6/6 fused ring system or an aldehyde group on ring B results in a significant loss of cytotoxic activity against HCT-15 cell lines [67].

#### Cytochalasin isolated from endophytic *Phomopsis* spp.

The coelomycetous genus *Phomopsis* belongs to the family Diaporthaceae and consists of approximately 900 fungal species from a wide range of hosts [68]. Endophytes beloging to the *Phomopsis* genus have attracted much attention in recent years for their ability to produce a variety of bioactive secondary metabolites as phytotoxins [69], and and other ones showing antimicrotubule, antimycobacterial, anticandida and cytotoxic activity [70, 71].

**Fig. 11:**
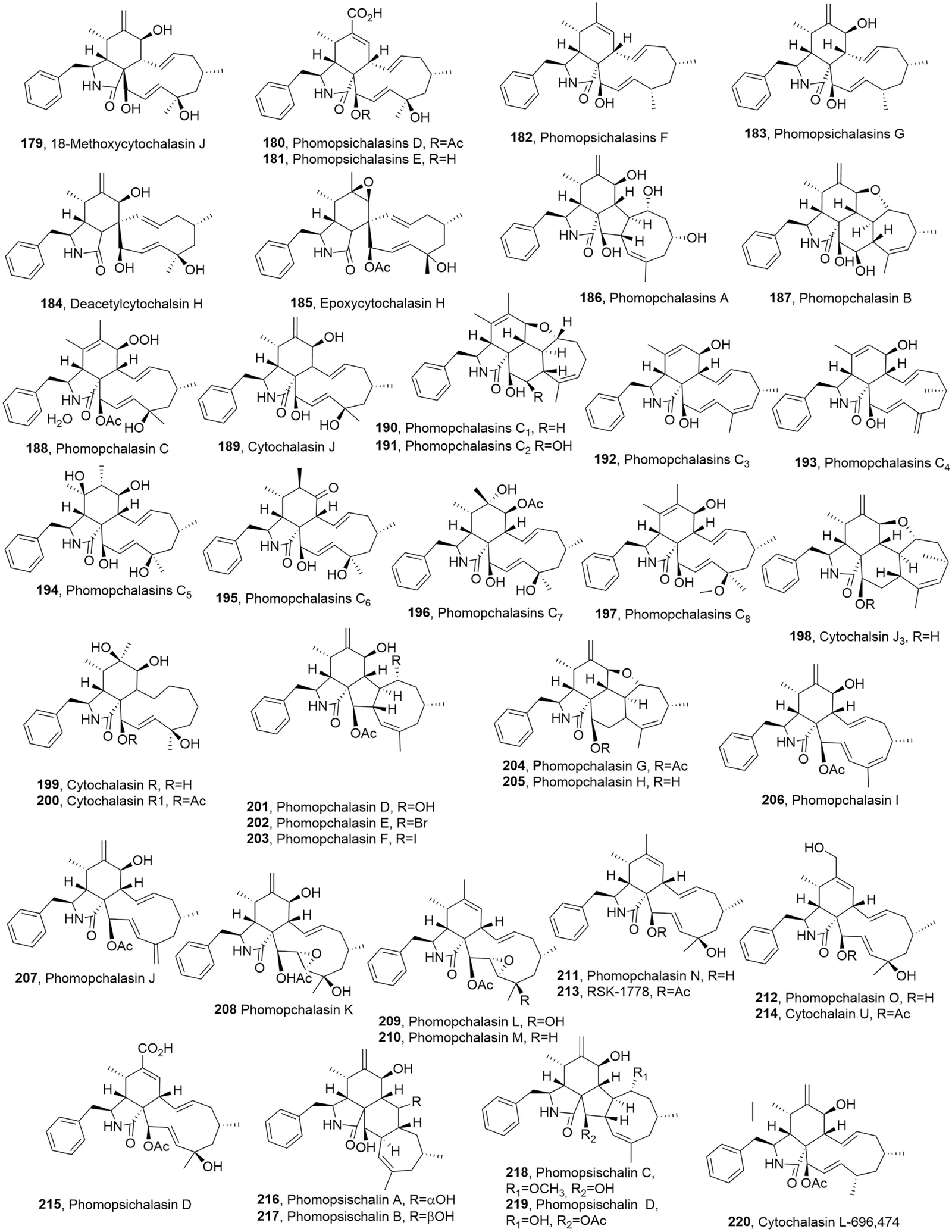
Cytochalasins isolated from the endophytic *Phomopsis* spp.

From the solid culture of the endophyte *Phomopsis* sp., 18-Methoxycytochalasin J (**179**, Fig. 11) was isolated together with the already known cytochalasin H, (22*E*, 24*S*)-cerevisterol, ergosterol, and nicotinic acid. All the compounds were tested for their cytotoxicity activity against human carcinoma cell line SMMC-7721 using fluorouracil, as a positive control and having an inhibition rate of 28.7% of SMMC-7721 cell line at 10 μg/mL. Compounds **179** and cytochalasin H inhibited the cancer cell growth by 24.4% at 10 μg/mL, and had an IC_50_ value of 15.0 μg/mL [72].

From different strains of the same endophyte fungus, namely *Phomopsis* spp. xy21 and xy22, both isolated from leaves of the mangrove, *Xylocarpus granatum*, phomopsichalasins D-G **(180**–**183**, Fig. 11), four new cytochalasins, were isolated together the well known phomopsischalasin G. In addition, from the same fungus were also isolated 4’-hydroxy-deacetyl-18-deoxycytochalasin H, deacetyl-18-deoxycytochalasin H, and 18-deoxycytochalasin H 21-*O*-deacetyl-l-696,474, deacetylcytochalasin H and epoxycytochalasin H (**184** and **185**, Fig. 11). Phomopsichalasins D and E (**180** and **181**) represented the first two 10-phenyl[11]-cytochalasans containing a 12-carboxyl function. All the compounds were assayed for cytotoxicity against eight human tumor cell lines (A375, AGS, HCT-8, HCT-8/T, A549, MDA-MB-231, SMMC-7721, and A2780) using cisplatin as the positive control. Phomopsichalasin G only exhibited potent activities against HCT-8, HCT-8/T, A549, MDAMB-231, and A2780 cell lines with IC_50_ values of 7.5, 8.6, 6.4, 3.4, and 7.1 μM, respectively [73].

Phomopchalasins A-C (**186**–**188**, Fig. 11) were isolated together cytochalasin J (1**89**, Fig. 11), from the endophytic fungus *Phomopsis* sp. shj2, which was obtained from *Isodon eriocalyx* var. *laxiflor*a. Compounds **186–189** were assayed for their cytotoxic activity, anti-inflammatory and antimigratory activities (using cisplatin, MG132 and cytochalasin D as positive control, respecively). Phomopchalasin C (**188**) showed moderate cytotoxicity against HL-60, SMMC-7721, and A-549 cell lines (IC_50_, 14.9, 22.7, and 21.1 μM, respectively) and inhibitory activity against NO production in LPS-activated RAW 264.7 macrophages with an IC50 value of 11.2 μM. In addition, Phomopchalasins A and B (**183** and **184**) exhibited an antimigratory effect against MDA-MB-231 in vitro with IC_50_ values of 19.1 and 12.7 μM, respectively [74].

From the endophytic fungus *Phomopsis* sp. xz-18., collected from the stems of *Cola acuminate,* phomopchalasins C1-C8 (**190**–**197**, Fig. 11) were isolated together with cytochalasins J3, R and R1 (**198–200**, Fig. 11) phomopchalasin B, cytochalasin H (**71**), cytochalasin J, cytochalasin N, cytochalasin O, cytochalasin P, and cytochalasin Q. All the cytochalasins were tested for their cytotoxicity, antipathogenic fungal and antibacterial activities. All compounds showed no toxicity against A375, A549, BGC-823, HCT116, HePG-2, HL-60, Jeko-1, Kyse-450, MCF-7 and U-2 OS (human melanoma, lung carcinoma, pancreatic colon hepatoma carcinoma acute promyelocytic leukemia, cell lymphoma, esophageal breast and osteosarcoma cancer cell lines). They were also inactive against the pathogenic fungi *Beauveria vuillemin*, *Curvularia lunata* and *Setosphaeria turcica*. Finally, in a disk diffusion assay, phomopchalasins C1, C3, and C4 (**190**, **192** and **193**) showed moderate antibacterial activity towards *B. subtilis* CMCC63501, *Bacillus pumilus* CMCC63202, *C. albicans* AS2.538, and *A. niger* ACCC3005 with inhibition diameters between 7 and 10 mm [75].

**Fig. 12:**
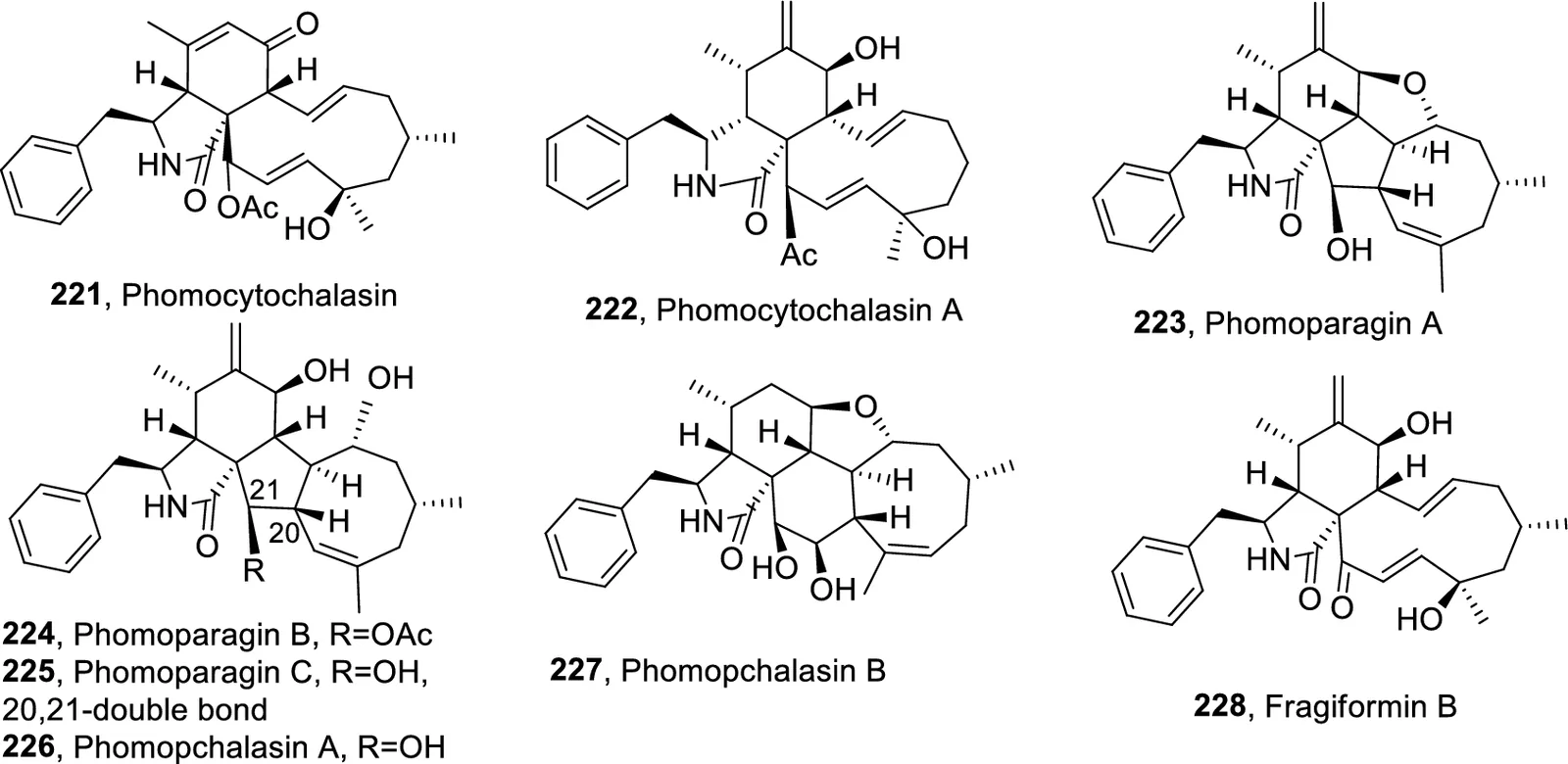
Cytochalasins isolated from *Phomopsis theicola* and *Phomopsis asparagi*

Phomopchalasins D-O (**201**–**212**, Fig. 11), including one brominated and one iodinated cytochalasins were isolated together with RKS-1778, cytochalasin U, phomopsichalasin D (**213–215**, Fig. 12) and cytochalasin J3 (**193**), cytochalasin J and cytochalasin H from the endophytic fungus *Phomopsis* sp. QYM-13. The fungus was collected from from healthy leaves of *Kandelia candel*. All the cytochalasins were assayed for their cytotoxic activity against the MDA-MB-435, HepG2, A549, and SNB19, MDA-MB-231 (breast, hepatocellular, lung, and breast carcinoma and glioblastoma) human cancer cell lines and Vero (African green monkey kidney) cell lines. Cytochalasins J and U and phomopchalain E exhibited cytotoxic activity towards some cancer cell line (IC_50_ values ranging from 4.9 to 8.2 μM). As expected cytochalasin H showed strong toxicity against MDA-MB-435 cell line (IC_50_value of 0.2 μM). The results also suggested that the acetyl group at C-21 have an important role comparing the activitiy of cytochalasin H, J, J3 and U, and RSK-1778. Finally, the brominated phomopchalasin E (**202**) showed a selective cytotoxicity against MDA-MB-435, which was six times stronger than that of the 7-hydroxylated analogue **204** [76].

From another strain of the same fungus, namely, *Phomopsis* sp. shj2, collected from the stems of *Isodon eriocalyx* var. *laxiflora*, phomopsischalins A-D (**216**–**219** Fig. 11) were isolated. These cytochalasins were tested for their in vitro antimigratory using cytochalasin D as positive control) and antimicrobial activities, but no effects was observed. Furthermore, the same compounds **216**–**219** were assayed for their abilities to induce lysosomal function. In fact, studing lysosomal biogenesis is a promising therapeutic strategy for the treatment of many human diseases as cancer and diabetic nephropathy. Phomopsischalasin B (**217**) noteworthy induced lysosomal degradation function during primary screening at a concentration of 20 μM and a dose-dependent effect was observed too. The same relation dose-function was showed in a LysoTracker Red uptake assay, which was conducted to determine lysosomal acidification and when confocal microscopy was performed to visualize the lysosome after LysoTracker Red staining [77].

Cytochalasin L-696,474 (**220**, Fig. 11) was isolated together with phomopsinin A-C, three polyketides, cis-hydroxymellein, and phomoxanthone A from the solid culture of *Phomopsis* sp. CAM212 [78], which is an endophytic fungus obtained from *Garcinia xanthochymus*. This plant, commonly known as false mangosteen, is a tropical fruit-yielding tree native of South East Asia, Polynesia, Africa, Australia, North Thailand, Myanmar, and Yunnan of China [79]. All compounds were tested on LPS-stimulated RAW 264.7 cells at the concentration of 10 µM for the measurement of NO production and cell viability. Cytochalasin L-696,474 (**220**) significantly inhibit nitric oxide production, but was highly cytotoxic to the treated cells. The latter compound (**220**), appeared to be a strong antiviral agent, due to its inhibitory effect on HIV-1 protease and had the NO inhibitory activity due to its cytotoxic effect on RAW 264.7 cells. As several cytochalasins inhibit cell division, and induce an apoptotic response via the inhibition of actin polymerization. The inhibitory effect of cytochalasin **220** on actin polymerization should be studied on different cell lines as its ability could be applied to a wide array of signaling pathways [78].

#### Cytochalasin from *Phomopsis theicola* and *Phomopsis asparagi*

**Fig. 13:**
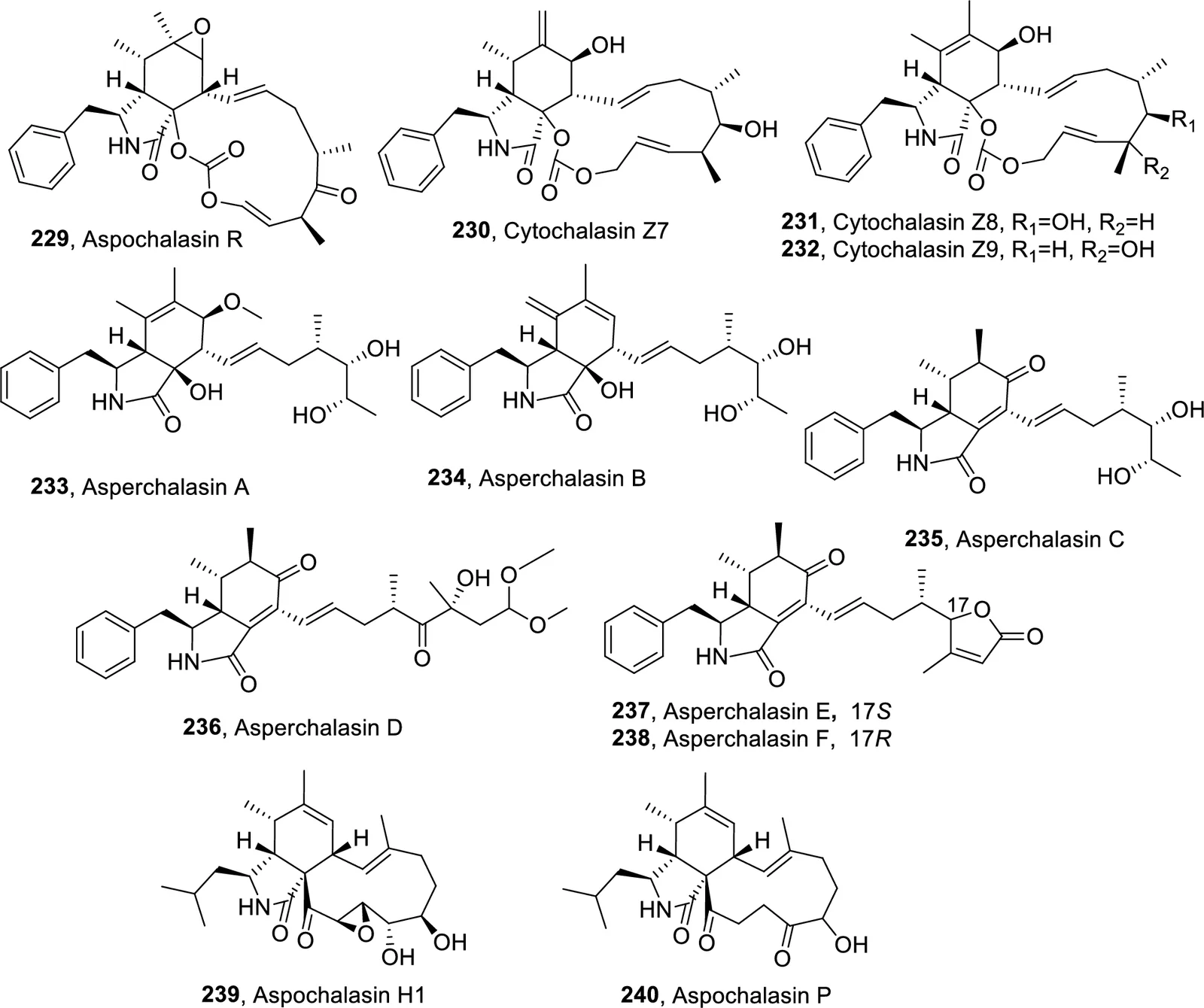
Cytochalasins isolated from *Aspergilu*s spp

From *Phomopsis theicola* BCRC 09F0213, an endophytic fungus collected from the leaves of the endemic plant *Litsea hypophaea* Hayata (Lauraceae), phomocytochalasin (**221**, Fig. 13), was isolated together with RKS-1778 (**208**), which was previously obtained *Phoma* sp. SNF1778 [80], cytochalasins H and N, dankasterone B, cyclo(L-Ile-l-Leu) from. The compounds obtained in sufficient amounts, were assayed for their inhibitory effects on LPS-induced nitric oxide synthase (NOS)-dependent NO and IL-6 production in the murine macrophage cell line RAW 264.7 and progesterone receptor antagonism. Cytochalasin N exhibited NO inhibitory activity compared to quercetin used as positive control (IC_50_ values of 77.8 and 36.8 ± 1.3 μM, respectively). Cytochalasin H exhibited progesterone receptor antagonism compared RU486 used as positive control (IC_50_ values of 1.42 μM and 0.063 nM, respectively). Cytochalasin N having a OH group at C-7 exhibited higher inhibition than its analog phomocytochalasin (**216**) with a carbonyl group at same carbon. This results showed the important role played by the substituent at C-7 to impart activity [81].

Phomocytochalasin A (**222**, Fig. 13) was isolated together with the already known cytochalasins H and J, phomoaspardiol, β-sitosterol, 2-phenethyl alcohol, tyrosol, *p*-hydroxybenzoic acid, *N*-(2-hydroxyphenethyl) acetamide, and (2*S*)-7-hydroxy-2-(2-hydroxypropyl)-5-methylchromone from *Phomopsis asparagi*, an endophytic fungus isolated from the Taiwanese endemic plant *Peperomia sui* T.T. Only cytochalasin J (**184**) exhibited androgen receptor (AR) antagonism against breast carcinoma MDA-MB-453 with a IC50 value of 6.20 μM [82]. From the same fungus, but collected from of the mangrove plant *Rhizophora mangle*, phomoparagins A-C (**223**–**225**, Fig. 12), three new cytochalasins, were isolated together with phomopchalasins A and B (**226** and **227**, Fig. 12), cytochalasins H and J and fragiformin B **(228**, Fig. 12). These compounds were tested for their inhibitory activity against concanavalin A (ConA)/lipopolysaccharide (LPS)-induced spleen lymphocyte proliferation and calcineurin (CN) enzyme. Cytochalasins H, phomoparagin B, fragiformin B and phomophalasins A and B showed interesting inhibitory activity associated to low toxicity. Furthermore, phomoparagin B (**224**) inhibited ConA-stimulated activation of NFAT1 dephosphorylation and block NFAT1 translocation in vitro and thus inhibiting the transcription of interleukin-2 (IL-2) [83].

#### Cytochalasins isolated from *Aspergillus* species

As above detailed reported fungi belonging to *Aspergillus* genus are wery well know as producers of secondary metabolites with different carbon keleton and biological activity. Among these there are also the ones the endophyte *Aspergillus* species.

From *Aspergillus* sp. CPCC 400735, collected from the medicinal plant *Kadsura longipedunculata*. Aspochalasin R (**229**, Fig. 13), a new cytochalasin, was isolated together with rosellichalasin, cytochalasins Z7, Z8, Z9 (**230**–**232**, Fig. 13), and cytochalasin Z17. From the same fungus were also obtained asperphenalenones A-E, aspulvinone R, aspulvinone Q, aspulvinone P, aspulvinone E, 3′,4′,4-trihydroxypulvinone, 3,4,4′-trihydroxypulvinone, aspernolide H, aspernolide I, aspernolide K, butyrolactone II, 2-O-methylbutyrolactone II, epicocconigrone A, epicoccolide B, 3-dihydro-4,6-dihydroxy-7-methylisobenzofuran, monomethylosoic acid, asterric acid, methyl-dichloroasterrate, sulochrin and dihydrogeodin. All the metabolites were preliminarly tested for their HIV-1 inhibition rates at 100 μM. The IC_50_ and CC_50_ (the 50% Cytotoxic Concentration) values of compounds with inhibition rates greater than 60% were then determined. In particular, asperphenalenones A and D, cytochalasin Z8 (**231**) and epicocconigrone A showed anti-HIV activities (IC_50_ values 4.5, 2.4, 9.2, and 6.6 μM, respectively, using lamivudine and efavirenz as positive control, IC_50_ values 0.1 μM and 0.4 × 10^−3^ μM, respectively) and CC_50_ values which were all greater than 100 μM [84].

Asperchalasins A-F (**229**–**238**, Fig. 13), six *seco*-cytochalasins, were isolated together with *seco*-cytochalasin E, cytochalasin E, rosellichalasin, and cytochalasin Z17 and asperlactones G-H from the solid cultures of the endophytic fungus *Aspergillus* sp. obtained from *Pinellia ternata* tubers. Asperchalasins E and F (**237** and **238**, Fig. 13) were intruing seco-cytochalasins possessing an α,β-unsaturated furanone moiety in their side-chains. All compounds were tested for their cytotoxicity against A-549 and MCF/DOX cell lines and showed cytotoxicity against A-549 cell line and, in particular, cytochalasin E exhibited obvious activity with an IC_50_ value of 7.8 μM. Asperchalasin E (**237**), at the concentration of 16 μM, also exerted a threefold enhancement of doxorubicin susceptibility on doxorubicin-resistant human breast cancer (MCF-7/DOX) cell line [85].

From endophytic fungus *Aspergillus* sp. FT1307, collected from the Hawaiian plant *Heliotropium* sp., aspochalasin H1, new cytochalasins, and aspochalasin P (**239** and **240**, Fig. 13), were isolated together with alatinone, dimerumic acid, NBRI 16716B (N,5-dihydroxy-N-(3–5-(3-(N-hydroxyacetamido)propyl)-3,6-dioxopiperazin-2yl)propyl)-3-methylpent-2-enamide), α-11-hydroxyurvularine, β-11-hydroxycurvularine, β-11-methoxycurvularine, 12-keto-10,11-dehydrocurvularine and WIN663. All the isolated compounds were assayed for their antibacterial activity against pathogenic *S. aureus* ATCC12600, *B. subtilis* ATCC6633 and methicillin-resistant *S. aureus* ATCC43300 (MRSA). Aspochalasin P (**240**), alatinone, 11-methoxy-curvularine and 12-keto-10,11-dehydro-curvularine exhibited activity in the range of 40 to 80 μg/mL. Previously SAR studies demonstrated that the keto unsaturation in the 11-membered macrolide ring and the stereochemistry of this latter were responsible for antibacterial activity of aspochalasins and in particular for that against Gram-positive pathogenic bacteria [86]. All the compounds were also tested for their antiproliferative activity against MCF-7 (human breast cancer cell line), T46D (human breast cancer cell line) and A2780 (human ovarian cancer cell line). Only aspochalasin P (**240**) and β -11-methoxycurvularine showed a mild antiproliferative activity against the A2780 ovarian cancer cell line (IC50 values of 17.29 µM and 11.76 µM, respectively) [86].

#### Cytochalasin isolated from *Aspergillus micronesiensis*

**Fig. 14:**
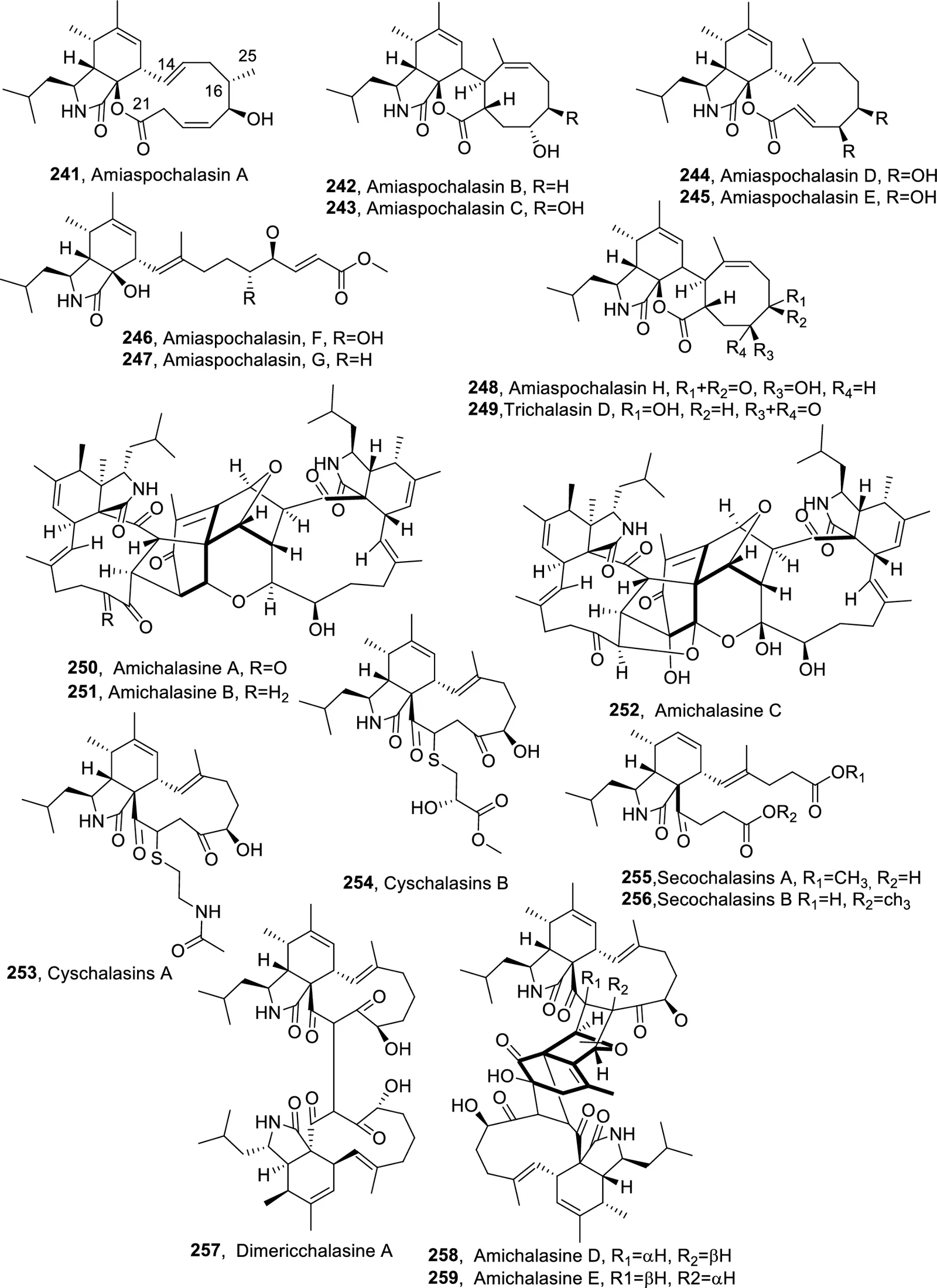
Cytochalasins isolated from *Aspergillus micron*esiensis

*Aspergillus micronesiensis*, derived from the root of the traditional Chinese medicinal plant *Phyllanthus glaucus*, produced several cytochalasin with diffrent carbon skeletons and biological properties. Amog them there are amiaspochalasins A-H (**241**–**248**, Fig. 14, eight undescribed aspchalasins, and trichalasin D (**249**, Fig. 14), a known analogue. Compounds **241**–**249** are aspochalasins with a C-21 ester carbonyl and the CH_3_-25 in compound **241** is located at C-16 rather than C-14 in respect to the previously reported aspochalasins. Compounds **242** and **243** are the first examples of aspochalasins with an unprecedented 5/6/6/8 tetracyclic ring system. Compounds **244** and **245** are diastereomers of aspochalasins I and J, respectively. Compounds **246** and **247** are the first aspochalasins featuring a long open-chain system. All the cytochalasins isolated were evaluated for their cytotoxicities against five human cancer cell lines (HL60, A549, Hep3B, U87, and SW480) and antimicrobial activities and compounds **244** and **245** showed, respectively, selective toxicity towards HL60 (IC_50_ 11.64 μM), and moderate antitumour activity against thefive cancer cell lines (IC_50_ values ranging from 6.18 to 22.04 μM). The other compounds **241**–**243** and **246**–**249** did not showed cytotoxic and antimicrobial activity [87]. From the same fungus amichalasines A-C (**250**–**252**, Fig. 14), a new type of cytochalasan heterotrimers, were isolated Compounds **250** and **251** possess an undecacyclic 5/6/11/5/5/6/6/5/11/6/5 ring system, and **252** has an additional tetrahydrofuran ring with a dodecacyclic 5/6/11/5/5/6/6/5/5/11/6/5 ring system. Compound were assayed towards six cancer cells showing potent cytotoxic activities through apoptosis induction mediated by caspase-3 activation and PARP degradation (IC_50_ values against HL60 cells were 1.71 and 3.74 μM, respectively) [88].

Cyschalasins A and B (2**53** and **254**, Fig. 14) and secochalasins A and B (**255** and **256**, Fig. 14), were isolated from the same endophytic fungus. Cyschalasins A and B (**253** and **254**) represent a new type of merocytochalasan featuring the fusion of an aspochalasin with a modified cysteine residue while secochalasins A and B are the first 17,18-seco-aspochalasins to be reported. Compounds **253** and **254** were cytotoxic and active against Gram-positive bacteria. Compounds **253**–**256** were tested for their in vitro cytotoxicity against five human cancer cell lines (HL60, A549, Hep3B, MCF-7, and SW480) and compounds **253** and **254** showed moderate activities against these cancer cells (IC_50_ values ranged from 3.0 to 19.9 μM) while compounds **255** and **256** were not toxic. Compounds **253** and **254** also exhibited antimicrobial effects against five bacteria (*E*. *coli*, *S. aure*us, MRSA, *Salmonella enterica*, *Pseudomonas aeruginosa*) and a fungus (*C. albicans*) and showed a significant antibiotic activity against MRSA (MIC_50_ values of 17.5 and 10.6 μg/mL and MIC_90_ values of 28.4 and 14.7 μg/mL, respectively [89]. From the same fungus three new cytochalasans namely dimericchalasine A and amichalasines D and E (**257**–**259**, Fig. 14), which are the first cytochalasan homodimer fused by a C-20/C-20′ single bond and a new type of cytochalasan heterotrimer with a decacyclic 5/6/11/5/5/6/5/12/6/5 ring system, respectively, were isolated.

The three cytochalasins **257**–**259** were bioassayed for their cytotoxic activity against leukemia cell lines. Compounds **257** and **258** showed moderate cytotoxic activities against HL60, THP-1, and U937 cells (IC50 values ranging from 5.11 to 12.9 μM). The same compounds induced an increased sub-G1 fraction cells when were investigated their cell cycle regulation activity in the significant G0/G1 cycle. Furthermore, the cytochalasin **258** induced apoptosis in HL60 when was used an Annexin V/PI staining kit [90].

#### Cytochalasin isolated from *Aspergillus spelaeus*

**Fig. 15:**
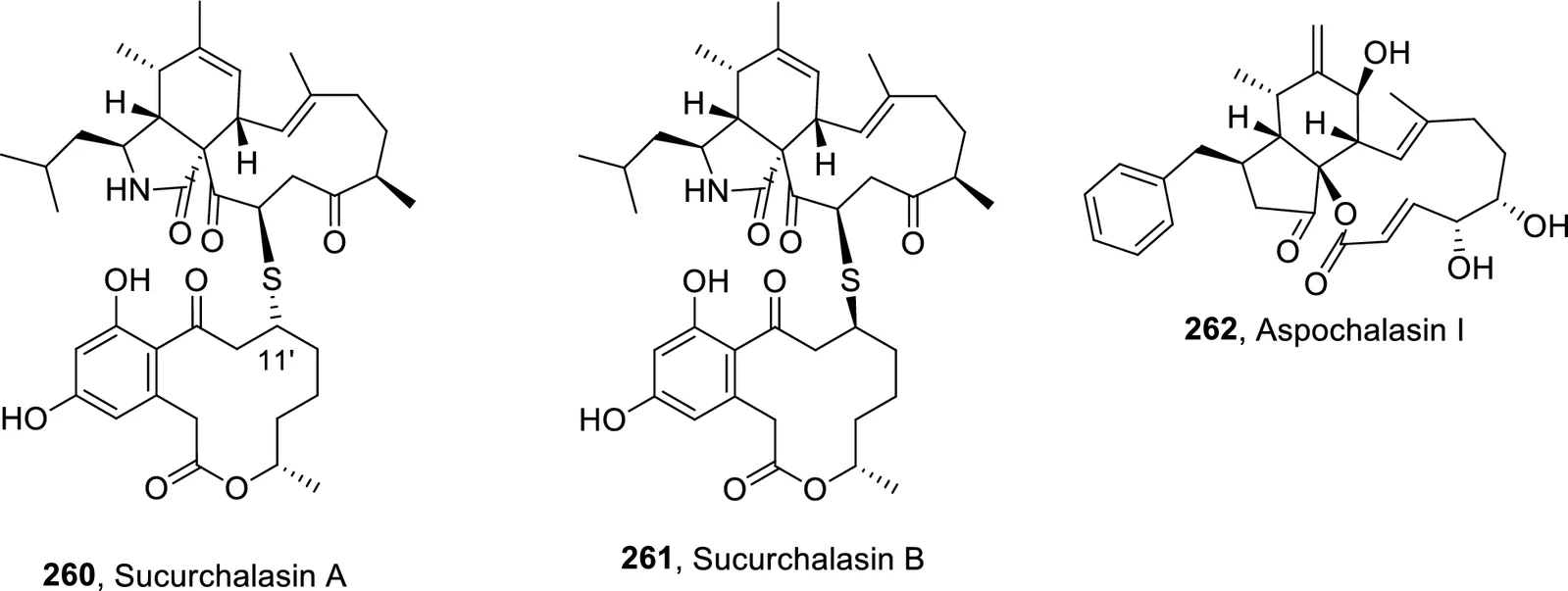
Cytochalasins isolated from *Aspergillus spelaeus91*

Sucurchalasins A and B (**260** and **261**, Fig. 15), two new sulfur-containing heterodimers of a cytochalasan and a macrolide, were isolated together with aspochalasin D (**32**), aspochalasin E (**33**), aspochalasin M (**35**), and aspochalasin I (**262**, Fig. 15), from the endophytic fungus *Aspergillus spelaeus* GDGJ-286. Furtermore, four macrolides, namely *trans*-dehydrocurvularin, βλ-dehydrocurvularin, 12-oxocurvularin, and 11-α-methoxycurvularin were also obtained. All compounds were tested for their cytotoxic activity against A2780, HeLa, SKOV3, and HepG2 cancer cell lines using cisplatin as a positive control. Sucurchalasins A and B (**260** and **261**) showed cytotoxicity against A278 (IC_50_ value of 3.9 and 8.3 μM, respectively) and *trans*-dehydrocurvularin exhibited cytotoxicity against A2780 and HeLa cells, (IC_50_ values of 4.3 and 7.9 μM, respectively). All other cytochalasins and macrolide did not showed toxicity (IC_50_ > 20 μM). These results suggested that the absolute configuration at the 11′-position in sucurchalasin A (**260**) and that the macrolide moiety contributes to their cytotoxicity were important for cytotoxicity against A2780, SKOV3, and HeLa cells. The compounds **260**, **261** and *trans*-dehydrocurvularin were also used to investigate the apoptosis after A2780 cells treatment and the compounds induced clear typical apoptotic morphological characteristics, in a dose-dependent manner. All the compounds were also tested for antibacterial activity toward seven bacteria including *Shigella dysentery*, *E. coli*, *Enterococcus faecalis*, *B. subtilis*, *Vibrio parahemolyticus*, *S. aureus*, and *Salmonella typhi*. Sucurchalasins A and B (**260** and **261**) showed antibacterial activity against *E. f*aecalis and *B. subtilis* (MIC values of 3.1 and 6.3 μg/mL, respectively). Aspochalasin D inhibited the growth of *E. faecalis* and *B. subtilis* (MIC value of 6.3 μg/mL.), while and *trans*-dehydrocurvularin inhibited *B. subtilis* and *S. dysentery* with MIC values of 3.1 and 6.3 μg/mL, respectively [91].

#### Cytochalasins isolated from endophyte fungi of Phoma genus

The genus *Phoma* has been studied for its ability to produce a wide range of secondary metabolites, associated with a range of bioactivities. The genus *Phoma* primarily includes *Phoma macrostoma*, *P. multirostrata*, *P. exigua*, *P. herbarum*, *P. betae*, *P. bellidis*, *P. medicaginis*, *P. tropica*, and many other species that are continually being identified for their potential secondary metabolites [91, 92].

**Fig. 16:**
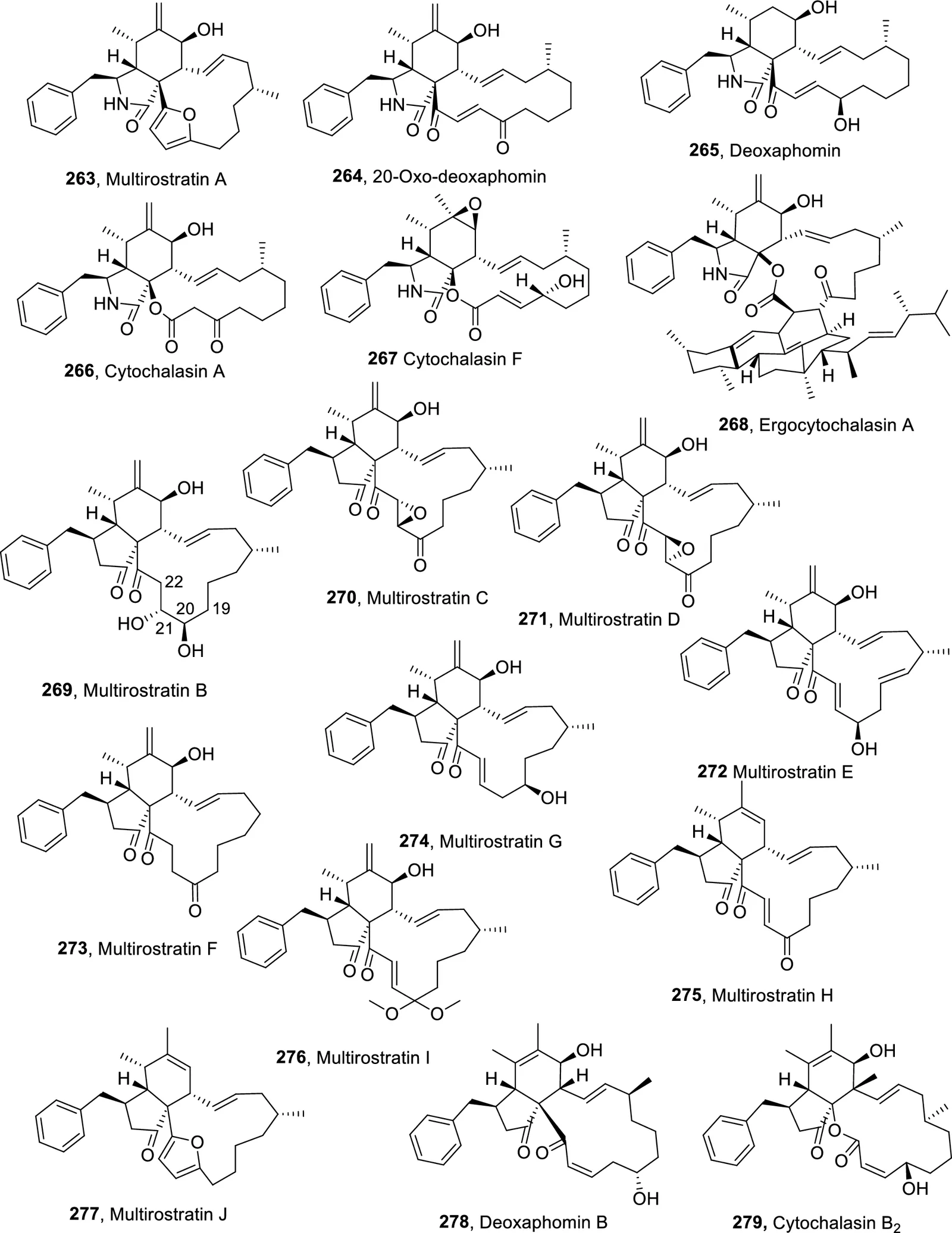
Cytochalasins isolated from *Phoma* spp

From the endophytic fungus *Phoma multirostrata* EA-12 which was obtained from fresh leaves of *Eupatorium adenophorum*. multirostratin A and 20-oxo-deoxaphomin (**263** and **264**, Fig. 16) were isolated together with the already known cytochalasin B (**77**), cytochalasin Z2 (**76**) and deoxaphomin, cytochalasin A, and cytochalasin F (**265**–**267**, Fig. 16), Cytochalasins **263** and **264** showed moderate cytotoxicity towards HL-60, A-549, SMMC-7721, MCF-7 and SW-480 tumor cell lines [92, 93]

Ergocytochalasin A (**268**, Fig. 16), a merocytochalasan, was isolated from the endophytic fungus *Phoma multirostrata* XJ-2-1, resident in the medicinal plant *Parasenecio albus*. Compound **268** tested for its cytotoxic activity against six cancer cell lines, namely HepG2 human hepatocellular carcinoma, A549 human lung carcinoma, MCF-7 human breast adenocarcinoma, HCT116 human colon carcinoma, HT-29 human colon carcinoma, and CT26 murine colon carcinoma cell lines, showed inhibitory effects with IC_50_ values ranging from 6.92 to 26.63 μM. Cytochalasin **268**, tested for its in vitro immunosuppressive activity, exhibited inhibition of 77.23% against ConA induced T cell proliferation at 20 μM and 102.65% against LPS-induced B cell proliferation at 10 μM. Furthermore, it showed antiviral activity against Human dengue virus type 3 (DV3), influenza A virus (H1N1) and respiratory syncytial virus (RSV) with EC_50_ values of 12.50, 40.92 and 6.49 μM, and the selectivity indexes (SI) of 1.82, 1.13 [93, 94]. Successively, from the same fungus multirostratin B-J (**269**–**277**, Fig. 16) were isolated together with cytochalasin Z2 (**76**), cytochalasin B (**77**), multirostratin A (**263**), 20-oxo-deoxaphomin (**258**), deoxaphomin (**259**), deoxaphomin B and cytochalasin B2 (**278** and **279**, Fig. 16). All cytochalasins were tested for their cytotoxicity against five cancer cell lines (MCF-7, HepG2, CT26, HT29 and A549 cell lines) along with one human normal cell line (LO2 cells) and using doxorubicin hydrochloride as a positive control. Multirostratin B, and E-J (**269** and **272**–**277**), and cytochalasin Z2 showed cytotoxic activity against parts or all of the five cancer cell lines with IC_50_ values ranging from 4.19 to 49.12 μM. Compounds 20-oxo-deoxaphomin, multirostratins A, D, G and H exhibited moderate selectivity to cancer cell lines. Multirostratin G and deoxaphomin inhibited CT26 and A549 (IC_50_ values of 6.03 and 5.04 μM, respectively) and MCF-7, HepG2, CT26, and A549 cell lines (IC_50_ values ranging 4.19–9.86 μM) respectively. Based on a preliminary SAR analysis, the HO-19 and HO-20 could be important structural features for this inhibitory activity. Multirostratins C and D having a 21,22-epoxide group were almost not toxic at a concentration of 50.0 μM suggesting that this epoxide group could induced a decrease of activity. Compound **274** tested for its influence on the cell cycles of CT26 and A549 cell lines, induced a significant G2-phase cell cycle arrest, suggesting that multirostratin G could cause cell shrinkages with no obvious apoptosis. Co-treatment with multirostratins A, H and J and cytochalasin Z2 of tumor related apoptosis inducing ligand (TRAIL) significantly decrease the A549 cell viability, showing that cytochalasins could probably be a new group of TRAIL sensitizers in lung cancer therapy [94–96].

#### Cytochalasins produced by endopyte fungi of D*iaporthe* genus

The genus *Diaporthe* (asexual state: *Phomopsis*) includes pathogenic, endophytic, and saprophytic species distributed in both temperate and tropical regions. These fungi have a broad host range, suggesting they move freely between hosts, frequently colonizing diseased or dead tissue, while some species are known to be host-specific [96, 97].

**Fig. 17:**
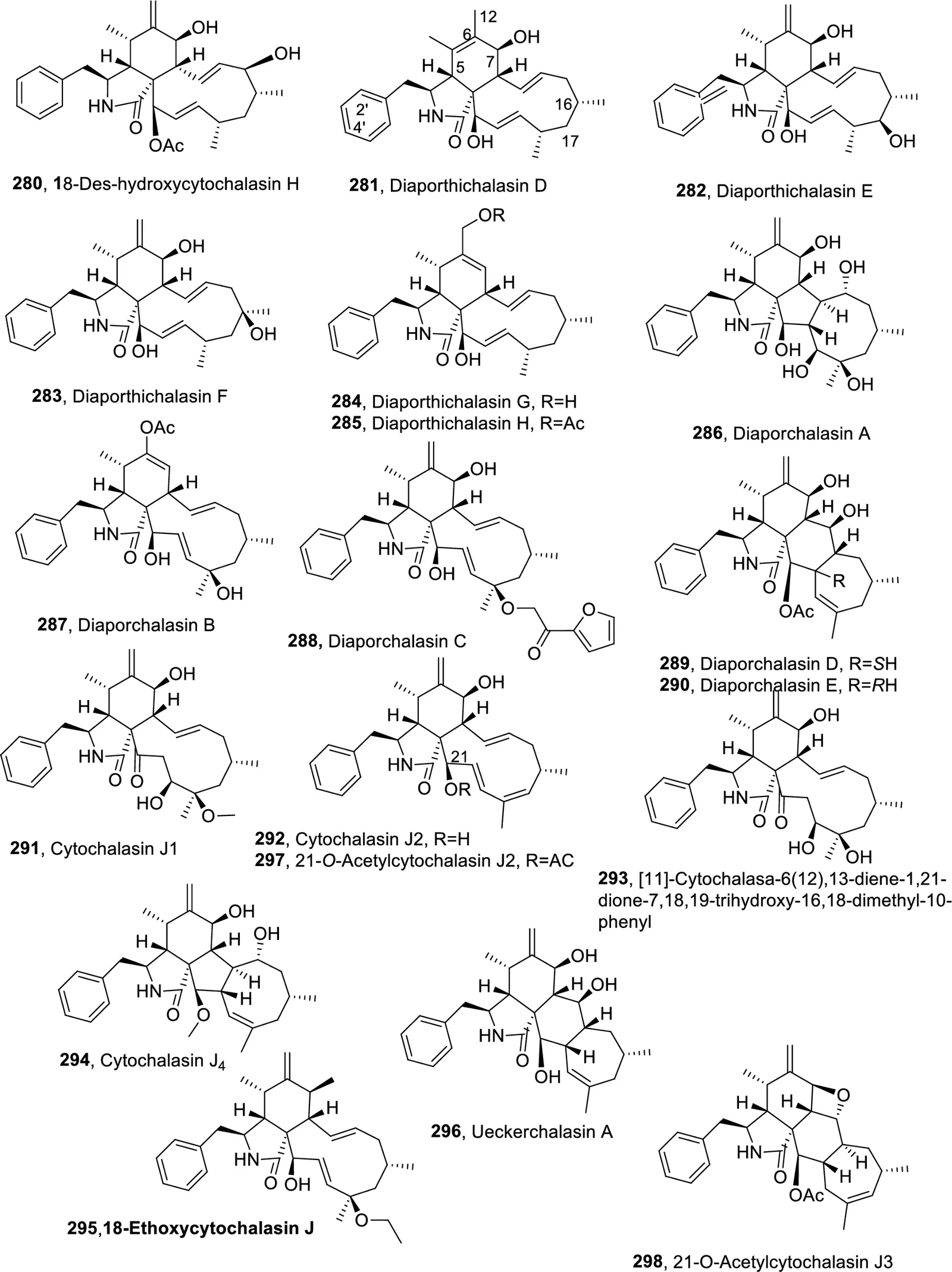
Cytochalasin isolated from *Diaporthe* spp

From the fungus *Diaporthe phaseolorum*-92C, which was obtained from the roots of *Combretum lanceolatum*, 18-Des-hydroxycytochalasin H (**280**, Fig. 17), a new cytochalasin, was Isolated from the fungal organic extract (20 μg/mL) exerted potent trypanocidal activity, reducing 82% of the number of amastigotes and trypomastigotes of *Trypanosoma cruzi*. Cytochalasin **280** killed 50% of *Schistosoma mansoni* adult worms when tested at 50 μg/mL and reduced the viability of *Leishmania amazonenses* promastigotes (IC_50_ 9.2 μg/mL) and of the cancer cells MDA-MB-231 and MCF-7 (IC_50_ = 17.5 and 8.88 μg/mL, respectively). In addition, the same cytochalasin **280** exhibited moderate antioxidant activity and showed cytotoxicity against normal cells GM07492A (IC_50_ 2049. μg/mL) [97, 98].

Diaporthichalasins D-H, (**281**–**285**, Fig. 17) were isolated from the solid cultures of the endophytic fungus *Diaporthe* sp. SC-J0138 obtained from the leaves of *Cyclosorus parasiticus*, which is a fern widely distributed in the southern part of China [99]. From the same fungus were also isolated, as above reported, diaporthichalasins A-C, phomopsichalasin G, and 21-*O*-deacetyl-l-696,474) [100]. All the cytochalasins were tested for their cytotoxic activity towards A549, Hela, HepG2, and MCF-7 cell lines. Diaporthichalasin H **(285**) showed cytotocicity against all tested cancer cell lines (IC_50_ values ranging from 9.9 to 32.1 μM), while cytochalasin G (**284**) had no toxicity (IC50 > 50.0 μM). Other compounds were shown to be selectively active against certain cell lines. In particular, they were all toxic toward HepG2 cells (IC50 = 8.8–38.1 μM) but inactive against MCF-7 cells; most of them, except diaporthichalasin E, **282**, showed toxicity against HeLa cell lines; diaporthechalasins C, D and 21-*O*-deacetyl-l-696,474, were still active against A549 cell lines (IC50 10.9–19.1 μM) [99]. These results suggested that the shift of C-6/C-12 double bond to C-5/C-6 enhanced the activity against HepG2 cells (21-*O*-deacetyl-l-696,474 vs. **281**) and that this simultaneous shifts of C-6/C-12 double bond and 7-OH to C-6/C-7 and C-12 respectively, led to the loss of activity (21-*O*-deacetyl-l-696,474 vs. **284**). Furthermore, the acetylation of the 12-OH recover the activity (**285** vs. 21-*O*-deacetyl-l-696,474). Other significant indication is that a hydroxylation of C-17, C-16, or C-2′-4′ determined the loss of the activity against A549 and/or HeLa cells (**282**, diaporthichalsin A and phomopsichalasin G vs. 21-*O*-deacetyl-l-696,474). All cytochalasins did not showed antibacterial activity against *S. aureus* and MRSA (MIC ≥ 50 μg/mL) [99].

From the endophytic fungus *Diaporthe* sp. BMX12, which was collected from the branches of *Aquilaria sinensis,* diaporchalasins A-E (**286–290**, Fig. 17) were isolated along with cytochalasin H, cytochalasin J, cytochalasins J1 and J2 (**291** and **292** Fig. 17), cytochalasin N, [11]-cytochalasa-6(12),13-diene-1,21-dione-7,18,19-trihydroxy-16,18-dimethyl-10-phenyl-(7S*,13E,16S*,18S*,19R*) and J4 (**293** and **294**, Fig. 17), fragiformin B, phomopchalasin A, phomoparagin B, phomopchalasin B, cytochalasin U, 18-ethoxycytochalasin J and ueckerchalasin A (**295** and **296**, Fig. 17). All cytochalasins were tested for their cytotoxicity towards five cancer cell lines including human leukaemia as K562, hepatoma, BEL-7402, gastric SGC-7901, human non-small cell lung A549, and cervical cancer HeLa cell lines. Cytochalasin **293** and fragiformin B showed strong cytotoxic activity (IC_50_ ranging from 4.4 to 47.4 μM). In the SAR point of view the results of cytotoxicity testes showed that the carbonyl group at C-21, was the characteristic group that impart toxicity to cytochalasins **293** and fragiformin B as well as the olefinic group Δ^19^, which appeared conjugated with this carbonyl C-21, could further improve the toxicity (**293** vs. fragiformin B) [100, 101].

21-acetoxy-cytocha J2 and 21-Acetoxy-cytochalasins J3 (**297** and **298**, Fig. 17), were isolated together with cytochalasin H (**71**), 7-acetoxycytochalasin H (**291**, endo diaporthe), cytochalasin J (**185**), cytochalasin J3 (**194**) and cytochalasin E (**108**) from *Diaporthe* sp. GDG-118, which is an endophytic fungus resident in *Sophora tonkinensis*. The antifungal activity of all the cytochalasins was tested against the plant path*ogentic* fungi such as *Alternaria oleracea*, *Pestalotiopsis theae*, *Colletotrichum capsici* and *Ceratocystis paradoxa* using carbendazim as positive control. Cytochalasin E showed significant antifungal activity against *A. oleracea*, *P. theae* and *C. capsici* with MIC values of 3.13, 1.56, and 1.56 μg/mL, respectively. Compound **297** also showed strong antifungal activity against *A. oleracea* and *P. theae* with MIC value of 6.25 μg/mL. Cytochalasin H exhibited potent activity against *A. oleracea*, *P. theae*, and *C. capsici* with MIC values of 6.25, 12.5, and 6.25 μg/mL, respectively. Only this latter cytochalasin exhibited activity against *C. paradoxa* with MIC value of 25 μg/mL, while the other compounds and positive control showed quite weak activity against the same fungus. The antibacterial activity of all the compounds was also evaluated and 21-acetoxycytochalasin J3 (**298**) showed moderate activity against *Bacillus anthraci* and *E. coli* with MIC value of 12.5 g/mL [101, 102].

#### Cytochalasins isolated from endophyte fungi belonging to other genera

**Fig. 18:**
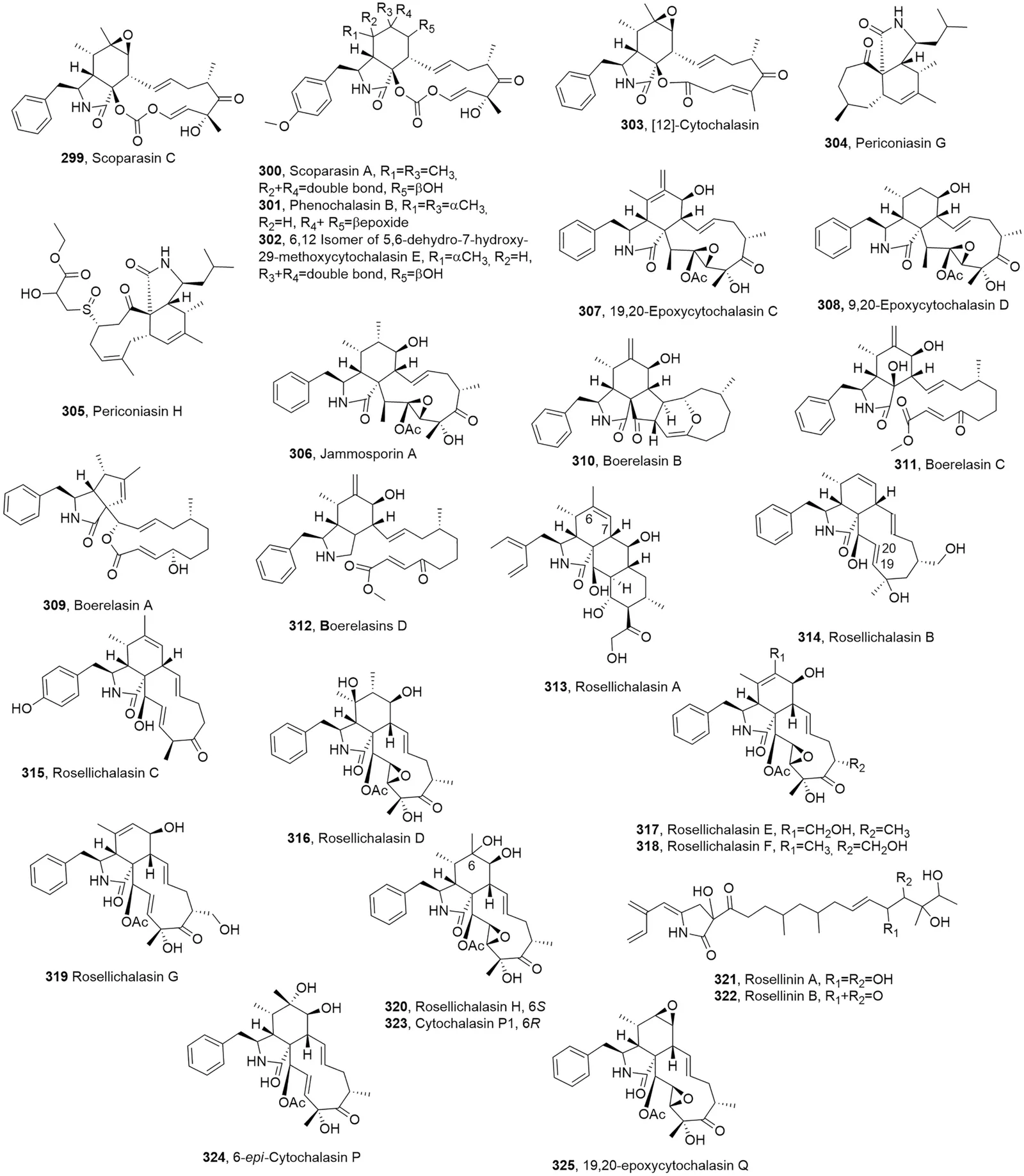
Cytochalasins isolated from other endophytic fungi

From the endophytic *fungus Eutypella scoparia,* collected from the leaves of *Hevea brasiliensis*, scoparasin C (**299**, Fig. 18), a new cytochalasin, was isolated together with the already known scoparasin A, phenochalasin B, 6,12 isomer of 5,6-dehydro-7-hydroxy-29-methoxycytochalasin E and [12]-cytochalasin 5,6-dehydro-7-hydroxy-29-methoxycytochalasin E (**300**–**303**, Fig. 18) and four known pimarane diterpene derivatives. All compounds were tested for their antibacterial (*S. aureus*), antifungal (clinical isolate of *Microsporum gypseum*), antimycobacterial (*Mycobacterium tuberculosis*), antimalarial (*Plasmodium falciparum*) and cytotoxic (KB and MCF-7 cell lines as well as African vervet monkey kidney fibroblasts, and Vero cells) activities, but none of them showed antimalarial activity. Phenochalasin B (**301**) showed mild activity against clinical isolate of *M. gypseum* with a MIC value > 200 μM. Scoporasins A and C, and [12]-cytochalasin (**300**, **299** and **303**) showed cytotoxicity against Vero cells (IC_50_ values ranging from 1.19 to 39.08 μM) and showed no other activity. Compound **294** exhibited potent cytotoxicity against KB cell lines (IC_50_ value of 2.46 mM) compared to that of the standard compounds doxorubicin and ellipticin. Compound **303** showed weaker activity (IC_50_ values of 80.48, μM). These results suggested that phenochalasin B (**301**) could be responsible for the cytotoxic activity shown by the crude fungal organic extract against KB cell lines. Finally, this latter cytochalasin and 6,12 isomer of 5,6-dehydro-7-hydroxy-29-methoxycytochalasin E (**303**) showed potent cytotoxicity against Vero cells (IC_50_ values ranging from 0.04 to 2.50 μM) [102, 103].

Periconiasins G and H (**304** and **305**, Fig. 18) were isolated from the endophytic fungus *Periconia* sp. F-31. Both compounds were tested for their cytotoxic and anti-HIV22 using camptothecin and efavirenz as positive control, respectively. Both periconiasins, tested at 10^–5^ M, showed no anticancer activity against five human cancer cell lines (A2780, HCT-8, Bel-7402, BGC 823, and A549), and compound **304** exhibited a moderate anti-HIV activity (IC_50_ value of 67.0 μM compared to that of efavirenz of 1.4 nM) [104, 105].

From the culture of the endophytic fungus *Rosellinia sanctae-cruciana*, collected from the leaves of *Albizia lebbeck* commonly known as the siris plant in the Indian Traditional Ayurvedic System of Medicine [103, 105] jammosporin A (**306**, Fig. 18) and cytochalasins C and D and their 19,20-epoxy derivatives (**307** and **308**, Fig. 18), were isolated The cytotoxic activity of the fungal organic extract and that of all cytochalasins was tested towards the human leukaemia cancer (MOLT-4) cell line and cytochalasin C showed considerable cytotoxicity with IC_50_ 6 μM60 μM/L, followed by compounds 307, 308, 306 and cytochalasin D with IC_50_ 8, 10, 20 and 25 µM respectively [103, 106].

From the endophytic fungus *Boeremia exigua*, obtained from the healthy leaf of *Fritillaria monatha* Migo, boerelasins A-D (**309**–**312**, Fig. 18), four new cytochalasins, were isolated and their cytotoxic activity was tested against five human cancer cell lines (HL-60, A549, SMMC 7721, MCF-7, and SW480) Boerelasins C (**311**) showed strong cytotoxicity against the HL-60, SMMC 7721, and MCF-7 cell lines (IC_50_ values of 2.89 ± 0.13, 4.33 ± 0.16, and 5.79 ± 0.07 μM, respectively), while boerelasins A and B (**309** and **310**) exhibited moderate cytotoxicity towards all of the five cancer cell lines (IC_50_ values ranging from 9.73 to 30.56 μM) [106, 107].

Rosellichalasins A-H (**313–318** Fig. 18) and rosellichalasins G and H as well as two new shunt metabolites, namely rosellinins A and B (**321** and **322**, Fig. 18) were isolated together with cytochalasins C, D, P and Q, 19,20-epoxycytochalasin C, 19,20-epoxycytochalasin D, cytochalasin P1, 6-epi-cytochalasin P, and 19,20-epoxycytochalasin Q (**323**–**325**, Fig. 18), from the endophytic fungus *Rosellinia* sp. Glinf021, collected from the medicinal plant *Glycyrrhiza inflata*. All cytochalasins were assayed for their cytotoxicity towards human cancer including HCT116, MDA-MB-231, BGC823, and PANC-1 cell lines. Most compounds showed moderate cytotoxicity against at least one cell line except rosellichalasins A-C and F (**313–315** and **318**) and rosellinins A and B (**321** and **322**). Cytochalasin Q showed a strong cytotoxic activity against all tested cell lines (IC_50_ values ranging from 0.6 to 6.4 μM.). Cytochalasins C and D and and their corresponding 19,20 epoxide analogues and 19,20-epoxycytochalasin Q showed good cytotoxic activities against HCT116 and BGC823. These results showed that the epoxide at C-6/C-7 was very an important feature for cytotoxicity and that the double bond at C-19/C-20 induced better cytotoxicity than the epoxy group [107, 108].

### Cytochalasins from fungi collected from marine organisms

Marine organisms, including fungi, bacteria, sponges, algae, molluscs, corals etc., is a real treasure to trove for finding secondary metabolites with original chemical structures and biological activities with potential applications in various fields [108–112].

**Fig. 19:**
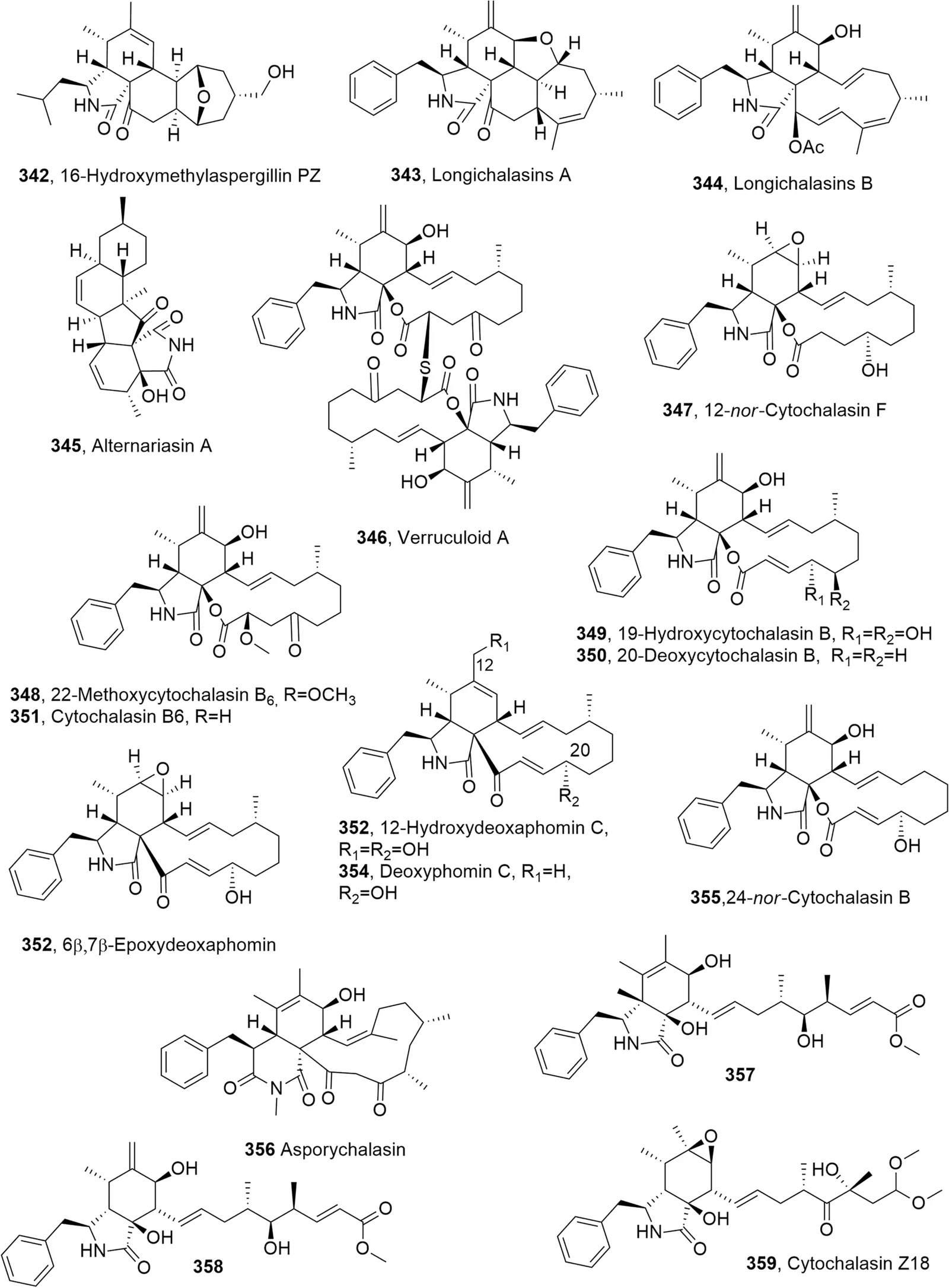

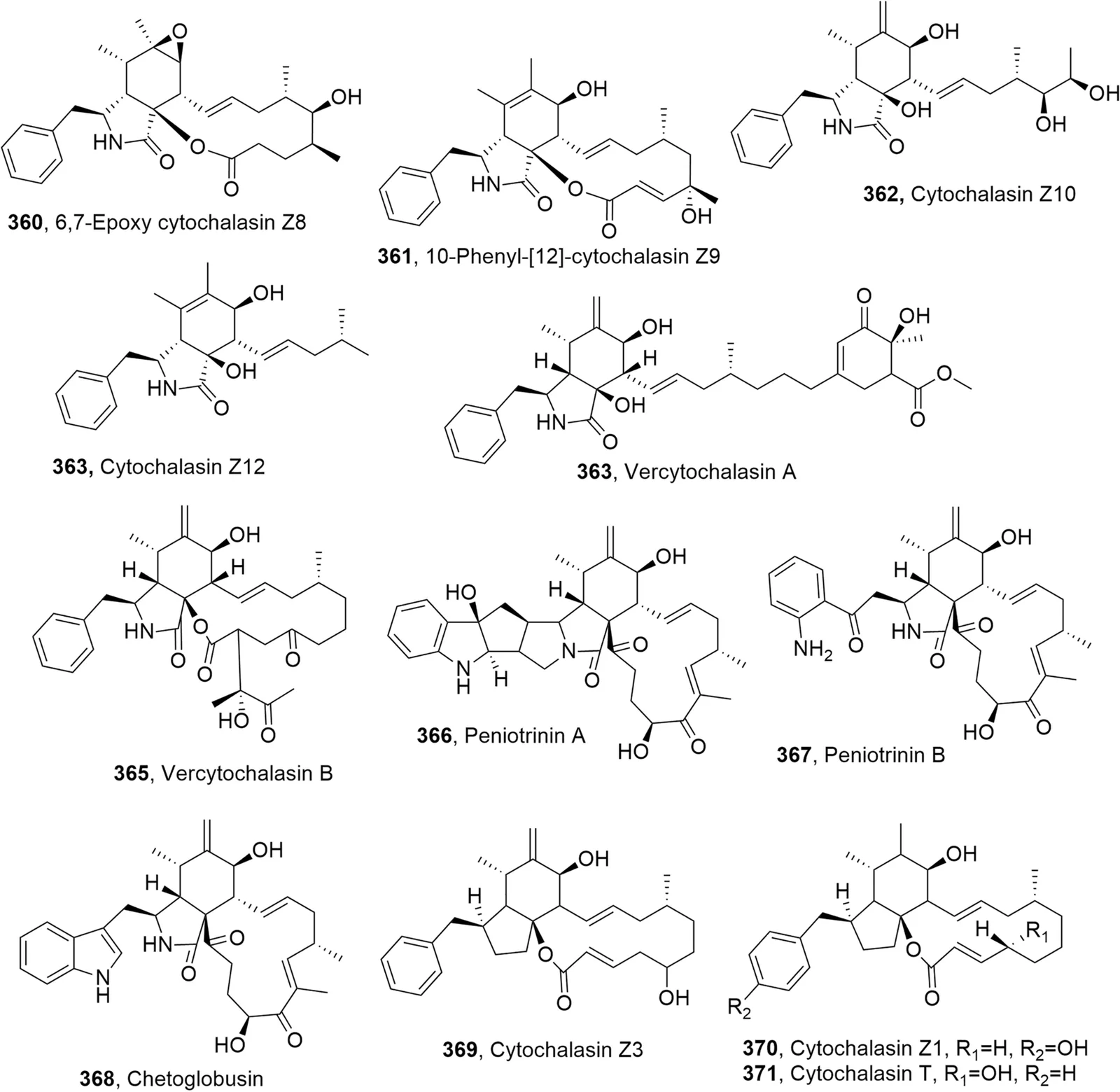
Cytochalasin isolated from endophite fungi of marine organisms

From the culture filtrates of *Aspergillus* sp., which was found in the gut of a marine isopod *Ligia oceanica*, aspochalsins V and W (**326** and **327**, Fig. 19), were isolated and tested against prostate cancer PC3 and the HCT116 cell lines. The two cytochalasins exhibited a moderate growth inhibitory activity (IC_50_ values of 30.4 and 39.2 μM, respectively). Considering also some previously reported data [110, 111], the methylthiol group should no effect the cytotoxicity of both aspochalasins [112, 113].

Diaporthalasin (**328**, Fig. 19) was isolated together with diaporthacol, *R*-mevalonolactone, dothiorelone C, (4*S*,7*S*,13*S*)-4,7-dihydroxy-1,3-tetradeca-1,5-dienolide, 4β-acetoxy-9β,10β,15 α-trihydroxyprobotrydial and *O*-methyldihydrobotrydial from the marine-derived fungus *Diaporthaceae* sp. PSU-SP2/4. When tested towards *S. aureus* and methicillin-resistant *S. aureus* diaporthalasin, showed significant antibacterial activity with a MIC value of 2 μg/mL [113, 114].

Scoparasin D (**329**, Fig. 19), a new cytochalasin, was isolated together with scoparasins A and C, and phenochalasin B (**292**), scopararane C and *ent*-4(15)-eudesmen-11-ol-1-one from the marine fungus *Eutypella scoparia*. The endophytic fungus was obtained from mangrove rhizosphere soil of Jimei, China. All compounds were tested for their cytotoxic activities against A375, A549, HepG2 and MCF-7 cell lines and only phenochalasin B showed strong inhibitory activity against all four cancer cell lines. The other compounds were not toxic (IC50 > 50μM [114, 115].

From the fungus *Arthrinium arundinis* ZSDS1-F3, collected from the sponge *Phakellia fusca*, arthriniumnins A-D **(330–333**, Fig. 19) and ketocytochalasin (**334**, Fig. 19) were isolated together with cytochalasins K, cytochalasin Z16 (**335**, Fig. 19), 10-phenyl-[12] -cytochalasin Z16, cytochalasin Z17 and rosellichalasin. Arthriniumnins A and B, and all cytochalasins were tested for their cytotoxicity against K562, A549, Huh-7, H1975, MCF-7, U937, BGC823, HL60, Hela, and MOLT-4 cell lines. Cytochalasin K showed moderate cytotoxic activity toward the K562, A549, Huh-7, H1975, HL60, Hela, and MOLT-4 cell lines (IC_50_ values ranging from 10.5 to 47.4 µM) while 10-phenyl-[12]-cytochalasin Z16 exhibited a moderate activity against K562, A549, H1975, MCF-7, U937, BGC823, HL60, Hela, and MOLT-4 cell lines (IC_50_ values ranging from 1.13 to 18.8 µM). The other compounds were not toxic against the cell lines tested (IC50 > 50 µM). When the all cytochalasins were tested for antituberculosis activity none of the compounds showed activity (MIC > 100 µM) [115, 116].

Cytochalasin H1 and H2 (**336** and **337**, Fig. 19) were isolated together with cytochalasins H, J, J1, J2 and J3 from Australian coastal marine sediment-derived fungus *Phomopsis* sp. (CMB M0042F). The last five compounds were previously reported as metabolites produced by different endophytic fungi. All cytochalasins were tested for their cytotoxic activity against a panel of human cancer cell lines including: SW620, colon adenocarcinoma; NCI-H460, lung carcinoma; HepG2, hepatocellular carcinoma; HEK, human embryonic kidney. Cytochalasins H and J have strong cytotoxicity, while the other ones, J1, J2, J3 H1 and H2 showed very significant, albeit variable and lower, levels of cytotoxicity [116, 117].

From marine-derived fungus *Xylaria* sp. SOF11 from the South China Sea, cytochalasin P, 19,20-epoxycytochalasin D, cytochalasin P1 and Zygosporin G were isolated together with 19,20-epoxycytochalasin N (**338**, Fig. 19) All the cytochalasins were assayed for their cytotoxic activity toward four tumor cell lines (SF-268, MCF-7, NCI-H460, and HepG-2) and exhibited strong cytotoxicity against two tumor cell lines MCF-7 and SF-268 (IC_50_ values varying between 0.33 and 4.17 μM) [117, 118].

Tricochalasin A and aspochalasin A2 (**339** and **340**, Fig. 19), two new cytochalasins, were isolated along with aspochalasins D, aspergilluchalasin and aspochalasins T from a different culture broth of *Aspergillus* sp., which was found in the gut of a marine isopod *Ligia oceanica*. All the compounds were assayed for their cytotoxicity against the prostate cancer cell line using doxorubicin (ADR) as positive control. Aspochalasins D exhibited strong activity against PC3 cell line, while other scompounds showed moderate activities [118, 119].

From the solid culture of *Westerdykella dispersa*, which were collected from a marine sediments, 16α-methylaspochalasin J and 16-hydroxymethylaspergillin PZ (**341** and **342**, Fig. 19), two new cytochalasins, were isolated and assayed for their antibacterial activity against *B. subtilis*, *Micrococcus luteus*, *Salmonella enterica*, *Proteus vulgaris*, *E. coli*, and *Enterobacter aerogenes*. Cytochalasin **341** showed moderate antibacterial activity against *B. subtilis* (MIC value of 50 μg/mL) while compound **342** exhibited activity against *P. vulgaris* and *E. aerogenes* (MIC value of 50 μg/mL) [119, 120].

Longichalasins A and B (**343** and **344**, Fig. 19), two new cytochalasins, were isolated together with cytochalasin H and cytochalasin J3, longidiacids A and B and two polyketides, from the deep-sea-derived fungus *Diaporthe longicolla* FS429. All the compounds were tested for their in vitro cytotoxicity against four human cancer cell lines (SF-268, MCF-7, HepG-2 and A549). Longichalasin B (**343**) exhibited antiproliferative effect against SF-268 (IC_50_ value of 16.44 μM, while cytochalasins H and J3 showed moderate activity at the concentration of 100 μM. Furthermore, all the compounds were also assayed against *Mycobacterium tuberculosis* protein tyrosine phosphataseB (MptpB) and only cytochalasin **344** and longidiacid A weakly inhibited at 35.4% the enzyme activity at a concentration of 50 μM [120, 121].

Alternariasin A (**345**, Fig. 19), a new cytochalasin, was isolated together with altertoxins I and II, tenuazonic acid, aloesol, and alternariol from the solid cultures of the Midui Glacier-derived fungus *Alternaria alternata*. All the compounds were assayed for antibacterial activity against *S. aureus* col (CGMCC 1.2465), *B. subtilis* (ATCC 6633), *S. pneumoniae* (CGMCC 1.1692), and *E. coli* (CGMCC 1.2340) using ampicillin and gentamicin as positive controls. Cytochalasin **345** displayed antibacterial activity toward the *S. aureus*, *B. subtilis* and *E. coli* (MIC values of 16.7, 16.7 and 33.3 μΜ, respectively) while ampicillin and gentamicin showed MIC values of 0.06, 1.28 and 2.09 μΜ, respectively. The other compounds proved to be inactive even at 80 μM. The same compounds were also tested for their cytotoxicity against five human tumour cell lines, Hela (cervical carcinoma), HepG2 (liver hepatocellular carcinoma), A549 (lung adenocarcinoma), MCF-7 (breast ade nocarcinoma), HCT116 (colorectal carcinoma) and one human glioma cell lines SHSY5Y and only altertoxin II exhibited cytotoxic activity against all the cell lines tested, (IC_50_ values of 6.3, 7.6, 12.7, 17.6, 5.4 and 6.3 µM, respectively, while the positive control cisplatin showed IC_50_ values of 14.1, 15.8, 17.9, 18.2, 9.5 and 12.7 µM). The other compound were not toxic at 50 µM [40].

Verruculoid A **(346**, Fig. 19), a cytochalasin dimer, 12-nor-cytochalasin F, 22-methoxycytochalasin B6, 19-hydroxycytochalasin B, and 20-deoxycytochalasin B (**347**–**350**, Fig. 19), which is a synthetic product obtained as a natural product for the first time, were isolated together with cytochalasin F, cytochalasin B6 (**351**, Fig. 19), cytochalasin B and deoxaphomin from *Curvularia verruculosa* CS-129. This is an endozoic fungus obtained from the inner fresh tissue of the deep-sea squat lobster *Shinkaia crosnieri*, which was collected from the cold seep area of the South China sea. All the compounds were tested for their antibacterial activities against aquatic pathogens, and verruculoid A (**346**) showed strong activity against *E coli* (MIC value 2 μg/mL) identical to that of chloromycetin used as control. 12-*nor*-cytochalasin F, 19-hydroxycytochalasin B and cytochalasin B inhibited the aquatic pathogenic bacteria *Edwardisella ictarda (*MIC value of 8 μg/mL while that of chloromycetin was 0.5 μg/mL) and *Vibro parahemolyticus* (MIC value of 4 μg/mL compared to that of chloromycetin which was 2 μg/mL, respectively). The MIC values of other compounds were larger than 32 μg/mL. These results suggested that the methyl group at C-6 (12-*nor*-cytochalasin F vs. cytochalasin F) and the hydroxy groups at C-19 and C-20 (20-deoxycytochalasin B vs. cytochalasin B and 19-hydroxycytochalasin B) could be structural features increasing the antibacterial activity. Furthermore, cytochalasins B and F, 19-hydroxycytochalasin B and 20-deoxycytochalasin B, which were the main metabolites, were also tested for their cytotoxicity against three tumor HCT-116, HepG-2, and MCF-7 cell lines. Cytochalasins B and F, and 19-hydroxycytochalasin B showed cytotoxic activity toward all tested cell lines with IC_50_ values ranging from 5.2 to 12 μM, while 20-deoxycytochalasin B was not toxic suggesting that the hydroxy groups at C-19 and C-20 significantly increased cytotoxicity (19-hydroxycytochalasin B and cytochalasin B vs. 20-deoxycytochalasin B) [121, 122].

Successively, from the same fungus 6β,7β-epoxydeoxaphomin C, 12-hydroxydeoxaphomin C, and 24-*nor*-cytochalasin B (**352**–**354**, Fig. 19), were isolated together with deoxaphomin C (**355**, Fig. 19) and proxiphomin. All cytochalasins were tested for their antibacterial activities against one human (*E. coli*) and nine aquatic pathogenic bacteria, namely *Aeromonas hydrophilia*, *E. ictarda*, *E. tarda*, *Micrococcus luteus, P. aeruginosa, Vibrio lginolyticus, V*. anguillarum, *V. harveyi*, and *V parahaemolyticus*), 6β,7β-Epoxydeoxaphomin C showed moderate antibacterial activity against aquatic pathogens *E. ictarda* and *E. tarda* (each with a MIC of 32 μg/mL), while deoxaphomin C exhibited strong activities against *E. coli* and *V. harveyi* (MIC of 2 and 8 μg/mL, respectively). Proxiphomin showed strong activities against *E. coli*, *V harveyi*, and *V. parahaemolyticus* (MIC values of 8, 4, and 1 μg/mL, respectively). These results suggested that the hydroxylation at C-20 reduced the antibacterial activities (24-*nor*-cytochalasin B vs. proxiphomin), while hydroxylation at both C-12 and C-20 induced the total loss of activity (12-hydroxydeoxaphomin C vs. 24-*nor*-cytochalasin B and proxiphomin) [122, 123].

Asporychalasin (**356**, Fig. 19), a new cytochalasin, was isolated from the marine fungus *Aspergillus oryzae*, collected from the Red Sea sediments collected off Jeddah, Saudi Arabia. Cytochalasin **356** exhibited moderate antiproliferative activity against three human cancer cell lines as lung carcinoma (A549), liver carcinoma (HepG2), and breast carcinoma (MCF7) (IC_50_ of 8.8 ± 0.4, 7.4 ± 0.2, and 8.3 ± 0.3 values μg/mL, respectively). When was tested asporychalasin (**356**) on *Zebrafish* embryos in vivo showed a LC_50_ (the concentration required to kill 50% of embryos) value, which was higher than 1 mg/mL, suggesting its selective activity on cancer cell lines [123, 124].

From coastal saline soil-derived fungus *Aspergillus flavipes* RD-13, two new Cytochalasins, named peniotrinins A and B, (**357** and **358**, Fig. 19), which were macrolactone ring opened derivatives of cytochalasin Z8 and Z7, respectively, were isolated together with cytochalasins Z18 (**359**, Fig. 19), of which was determined the absolute configuration, cytochalasins E, Z7 and Z8, 6,7-epoxycytochalasin Z8 (**360**, Fig. 19), cytochalasin Z13, 6,7-epoxycytochalasin Z8, 10-phenyl-[12]-cytochalasin Z9, cytochalasin Z10 and cytochalasin Z12 (**360**–**363**, Fig. 19) All cytochalasins were assayed for their cytotoxic activities against NSCLC cells A549, H1299 and H520. Cytochalasins E, Z7, Z8 and 6,7-epoxycytochalasin Z8 were cytotoxic to all these three cancer cells with cytochalasin E being the strongest inhibitor with IC_50_ values ranging from 0.15 to 0.43 μg/mL. 6,7-Epoxycytochalasin Z8 exhibited significant cytotoxicities toward A549 and H520 (IC_50_ values of 4.8 and 0.6 μg/mL) while cytochalasins Z7 and Z8 were found to be less active and seco-cytochalasin 358 and 10-phenyl-[12]-cytochalasin Z9 showed weak inhibitions toward A549 and H520 (IC_50_ values ranging from 21.7 to 40.4 μg/mL). Cytochalasin Z10 only showed cytotoxicity against H520 with the IC50 value of 21.0 μg/mL. However, compounds **357**, Cytochalsin Z12, Z13 and Z18, were noncytotoxic against all these three selected cells. These results suggested that the open chain could decrease the cytotoxicities sharply when comparing the cytotoxic data of compounds **357** and cytochalasin Z7, as well as cytochalasin E and Z18 [124, 125].

Vercytochalsins A and B (**364** and **365**, Fig. 19) were isolated from *C. verruculosa* above cited as producer of other cytochalasins. Both cytochalasins **364** and **365** were tested for their angiotensin-I-onverting enzyme (ACE) inhibition using captopril as positive control (IC_50_ 15.0 nmol/L), and compound **364** appeared to be a strongest inhibitor (IC_50_ value 505 nmol/L) compared to the positive control. ACE is regarded as a key enzyme that regulates blood pressure and translates angiotensin I into an-giotensin II in the human circulating or endocrine system. In the antibacterial screening, cytochalasin **365** had potent inhibitory activity against aquatic pathogenic bacteria *V. anguillarum*, *V. harveyi*, and *V. parahaemolyticus* (MIC values 32, 8 and 4 μg/mL, respectively). These results suggested that compound **365** has potential to be a broad-spectrum antibiotic against aquatic pathogenic bacteria in the aquapathogens of the genus *Vibrio* [125, 126].

From the solid culture of *Peniophora* sp. SCSIO41203, isolate from a soft coral sample near Weizhou Island (Guangxi, Beihai, China), peniotrinins A and B (**366** and **367**, Fig. 19), a chetoglobusin (**368**, Fig. 19), peniotrinins C-E and peniotrinin F, citrinine tetramic acid derivatives, were isolated together with dicitrinin A, phenol A acid, dihydrocitrinone, dihydrocitrinin, (3*S*, 4*S*) sclerotinin, tumonoic acids K and L, and alteamide Cytochalasins **366** and **367** and the chetotoglobusin **(368)** were tested for their anti-prostate cancer activity using 22Rv1 cell lines, which are PCa cells that express the androgen receptor. Peniotrinins A and B (**366** and **367**), showed weak activity at 10 µM, peniotrinin C inhibited PC-3 cell viability (IC_50_ of 16.58 ± 0.25 µM). Compound **368** also inhibited cell viability of another PCa cell (DU145), which does not express an androgen receptor (IC_50_ of 55.92 ± 2.35 µM). Finally, the same chetoglobusin tested on the cell viability of HeLa, a cervical cancer cell, showed a reduced activity (IC_50_ of 75.51 ± 3.58 µM) [126, 127].

### Cytochalasins from other sources

The results reported in the previous three sections confirmed those reported by Skellam [10] on the almost exlusive fungal origin of cytochalasains. In fact, from in deep literature research there are not cytochalasins produced by plant, bacteria, insects or other living organisms at leasts in the last decade. The only exception was the isolation of cytochalasin E from *Pleurosticta acetabulum*, a lichen widely present in Europe. *P. acetabulum* is a common lichen growing on nutrient-rich bark from trunks of broad-leaved trees, *Ulmus*, *Fraxinus*, *Acer pseudoplatanus* and *Sambucus*. *P. acetabulum* had a significantc toxicity against HT-29 colorectal cancer cells, which is due to the presence of cytochalasin E. (IC_50_ value of 10 μM). In particular, cytochalasin E specifically inhibited endothelial cell proliferation in vitro and angiogenesis and tumor growth in vivo and when tested on other cancer cell lines (A549, HeLa, BEL-7402, RKO) showed a decreased activity (IC_50_ ranging between 37 and 64 μM). Thus, HT-29 cells appeared to be particularly sensitive to cytotoxic activity of this cytochalasin [127, 128].

## Some biotechnological applications of cytochalasins

Some cytochalasins have shown potential promising activity for applications in agriculture for weed control as biopesticides, and in medicine, as drugs for the treatment of infectious and noninfectious diseases.

Different strains of *Phoma exigua* var. *exigua*, collected in Russia and Norway and proposed as mycoherbicides for the biocontrol *Sonchus arvensis* L. (commonly called perennial sowthistle) produced cytochalasins*. S. arvensis* L. is a weed that grows vigorously and forms dense patches and easily spreads for the prolific seed production to infest new area. This plant species is considered to be an important weed in Europe and North America as it infests many habitats such as cultivated fields, roadsides, pastures, rangelands, railway embankments, and lawns. When grown in liquid and solid cultures, *P. exigua* var. *exigua* produced *p*-hydroxybenzaldehyde, cytochalasins B, F, Z2, deoxaphomin and cytochalasin Z3 (**369**, Fig. 19). Cytochalasins Z2 and Z3 were previously isolated together with the well known cytochalasins B, F, deoxaphomin and cytochalasins Z1 and T (**370** and **371**, Fig. 19), from a wheat solid culture of *Pyrenophora semeniperda*, a pathogen proposed for the biological control of grass weed *Bromus tectorum*. *B. tectorum* (cheatgrass or downy brome) is an exotic winter annual grass weed that causes serious losses in intensive agriculture, particularly in winter cereal crops and is also a major problem on rangelands in the western U.S.A. *P. semeniperda*, proposed as a potential mycoherbicide for the control of this weed, produced on solid wheat seed cultures all the above cited cytochalasins, except for cytochalasins Z1 and Z2 [128, 129]. Cytochalasins Z1, Z2 and Z3, inhibited the germination of wheat and tomato seedlings in comparison with the other above cited cytochalasins and the 21,22-dihydroderivative of cytochalasin B. Assayed on the leaves of both *C. arvense* L. and *S. arvensis* L., deoxaphomin demonstrated the highest level of toxicity on leaves of *S. arvensis* L. Cytochalasin Z2 appeared to be the least toxic cytochalasan on both plants due to the lack of the secondary hydroxy group on C-7, which in the previous SAR study appeared to be one of the most important structural features to impart phytotoxicity [129, 130].

The potential biotechnological applications of cytochalasins in medicine were extensively described in the Sect. 1–3. Essentially cytochalasins possess a potential anticancer and antibiotic activity.

Cytochalasins acts against microfilaments and are used in basic research to understand cytoskeletal mechanisms. However, as previously reported, they possess strong antitumor activity due to their ability to preferentially damage malignant cells, as demonstrated by their minimal effects on normal epithelial and immune cells. Furthermore, cytochalasins also influence the final stages of mitosis, suggesting their potential use in combination with microtubule-targeting agents to elicit a profound synergistic effect on malignant cells. Therefore, cytochalasins could be used to supplement current chemotherapy therapies to improve their efficacy and reduce the prevalence of drug resistance in the clinical setting [11]. To study these synergistic effects, the cell lines EL4 (H2b T-cell lymphoma), P815 (H2d mastacytoma), Z2B (transformed human Epstein-Barr virus), CH33 (H2k B-cell lymphoma), and LS102.9 (H2s B-cell lymphoma) were exposed to treatments with cytochalasin B alone, vincristine alone, and cytochalasin B/vincristine combined. All cell lines showed the highest rate of DNA fragmentation with the cytochalasin B/vincristine treatment. This rate was in fact greater than the sum of the DNA fragmentation caused by both compounds when applied alone, supporting the hypothesis of their synergistic action. Therefore, this methodology could represent a new chemotherapy approach [11].

An alternative methodology to combat cancer diseases could be the use of cytotoxic cytochalasins associated to sonodynamic therapy (SDT). This latter is an ultrasound-based method in which chemotherapeutic agents known as sonosensitizers are administered to enhance the effectiveness of ultrasound's preferential damage to tumor cells. Cytochalasin B is a cytokinesis inhibitor that preferentially increases and multinucleates malignant cells, making them much more sensitive to ultrasound irradiation. Cytochalasin B preferentially damaged U937 human blood/leukemic cells both before and after sonication and also reduced rapid proliferation because the clonogenicity of U937 cells was significantly reduced after treatment. Therefore, cytochalasin B may have valuable therapeutic applications when combined with SDT [130, 131].

As reported in some cases in Sects. 1–3, cytochalasins have differentiated antibacterial activity against different microbial pathogens among these, *Shigella flexneri* SDINT was the most sensitive bacterium, and *Vibrio cholerae* SG24 and *V. cholerae* PC2 were the most resistant ones. The well-known antibiotic ampicillin showed no toxicity against these three bacteria at concentrations up to 512 μg/mL. Some cytochalasins were selectively effective against these multidrug-resistant bacteria. In particular 18-methoxycytochalasin J, cytochalasins H and J showed inhibitory effects on 4/6 (66.66%), 5/6 (83.33%), and 4/6 (66.66%), respectively, of the studied microorganisms. The observed MBC (Minimal Antibacterial Concentration) values indicated that most of them corresponded to the MIC values. Thus, cytochalasins have potential medicinal application against MDR enteropathogenic bacteria, including clinical isolates of toxigenic *V. cholerae*, the causative agent of the deadly disease cholera, and *Shigella* sp., the agent responsible of shigellosis. Furthermore, significant antibacterial activity was also observed against *S. aureus* [131, 132].

## Conclusions

The review reports the advances on chemistry, biological activities, mode of action and structure–activity relationships of new cytochalasins isolated in the last decade (2014–2024). In particular, the content of about hundreds articles published on this topic and found by Sci-Finder Google Scholar, Shi-hub research was reported focusing the attention on the chemitry and biological properties of new cytochalasins isolated from terrestrial, endophytic and likenes marine fungi. The results of several SAR studies were also described as well as the advances potential biotechnological applications of some cytochalasins in agriculture and medicine. Considering the large number of fungi, marine organisms, and other organisms distributed across the underwater world that have not yet been studied, and those known to be able to adapt to their surrounding environment by essentially changing their secondary metabolism, many more cytochalasins could be isolated and studied for their chemical and biological properties. The discovery of cytochalasins with new carbon skeletons is very likely to increase interest in this class of natural compounds for their potential applications as above reported.

## Data Availability

Data sharing is not applicable to this article, as no datasets were generated or analysed during the current study; all the articles analysed in this study are cited with its DOI (when available).
